# Molybdenum Cofactor Model Reveals Remarkable Redox Activity at Both Molybdenum and the Pyranopterin Dithiolene Ligand

**DOI:** 10.1021/jacs.4c17577

**Published:** 2025-04-28

**Authors:** Jinming Liu, Angelina Rogatch, Benjamin R. Williams, Chelsea Freer, Chiara Zuccoli, Jing Yang, Martin L. Kirk, Sharon J. Nieter Burgmayer

**Affiliations:** a Department of Chemistry, Bryn Mawr College, Bryn Mawr, Pennsylvania 19010, United States; b Department of Chemistry and Chemical Biology, The University of New Mexico, MSC03 2060, 1 University of New Mexico, Albuquerque, New Mexico 87131-0001, United States

## Abstract

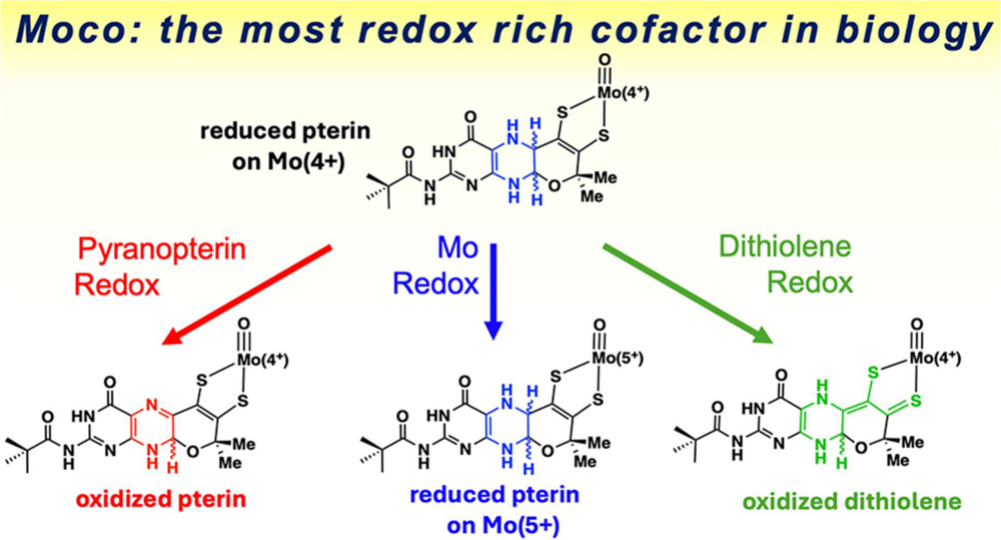

The molybdenum (Moco) and tungsten (Tuco) cofactors are uniquely found in pyranopterin dithiolene (PDT) molybdenum and tungsten enzymes, yet the roles of this electronically complex PDT ligand in the catalytic cycles of these enzymes has yet to be revealed. After more than a decade of effort, we have synthesized and characterized a model compound containing a reduced PDT ligand coordinated to a diamagnetic d^2^ low-spin Mo(4+) ion, mimicking the MoO(PDT) structure common to most Mo enzyme active sites. A combination of 1D and 2D NMR spectroscopies, augmented by molecular geometry optimization computations, confirms that both *R,R-* and *S,S-*diastereomers coexist in the synthetic final product. Redox processes at both the Mo ion and the pyranopterin are detected by cyclic voltammetry. The two-electron oxidant DCIP oxidizes the pterin component of the ligand in methanol, whereas no reaction occurs in aprotic acetonitrile. Addition of 1 equiv of the one-electron oxidant Fc^+^ stoichiometrically oxidizes the Mo(4+) ion to the paramagnetic d^1^ Mo(5+) species, a result supported by electron paramagnetic resonance (EPR) spectroscopy. However, the addition of more than 1 equiv of Fc^+^ results in oxidation of the reduced pyranopterin to yield a Mo(4+) complex of the oxidized pyranopterin dithiolene ligand, a result supported by both the cyclic voltammetry and electronic absorption titrations. The concrete examples from these model studies suggest how the unique electronic structure of the PDT ligand in Moco and Tuco may enable variable redox reactivity in enzymatic catalysis, highlighting its role as a complex noninnocent biological ligand.

## Introduction

**Figure 1:**
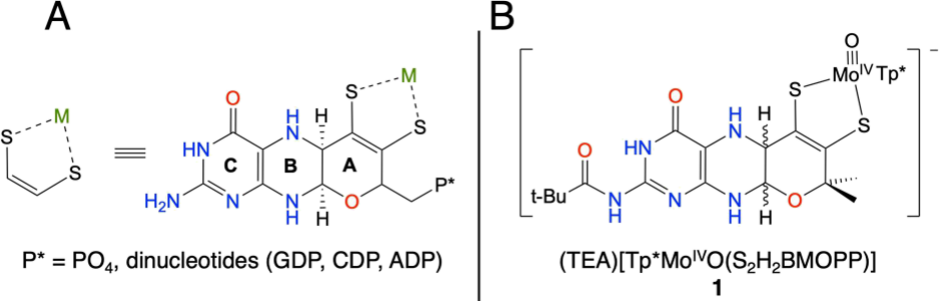
(A) Structure of the pyranopterin dithiolene ligand (PDT) in Mo and W enzymes. A = pyran ring, B = pyrazine ring, C = pyrimidine ring, pterin = B + C rings. (B) New synthetic pyranopterin dithiolene model (**1**) for Moco.

The pyranopterin dithiolene ligand (PDT) is uniquely found in all molybdenum and tungsten enzymes, with the sole exception being the Mo-containing nitrogenase.^1−12^ The PDT is one of several ligand structures in biology that have endured through 2.5 billion years of evolution, originating in the first life form known as the last universal common ancestor (LUCA).^13^ However, in the contemporary biosphere, the PDT is predominantly found in the more numerous pyranopterin molybdenum enzymes.^10^ The cofactors containing PDT and either Mo or W ions are known as the molybdenum cofactor (Moco) and tungsten cofactor (Tuco), respectively.^3,14^Figure 1A and 1B provide structures for the PDT ligand and the [Tp*MoO(S_2_H_2_BMOPP)]^1–^ (**1**) complex that closely models the basic MoO(PDT) fragment found in Moco.

**Figure 2:**
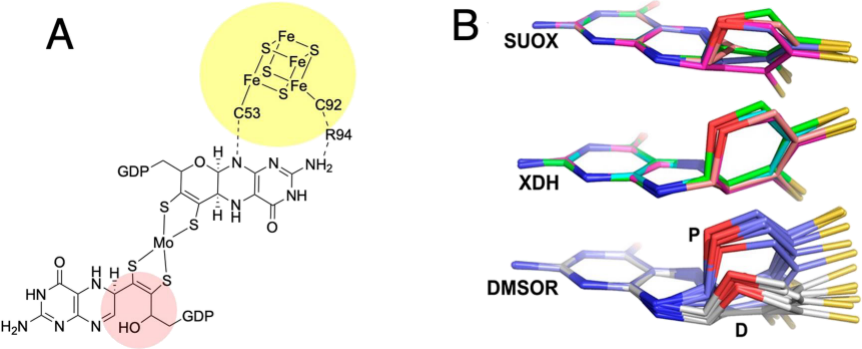
(A) Moco in *E. coli* nitrate reductase NapGHI (PDB: 1Q16) exhibits a H-bonding interaction with a FeS cluster (yellow circle). Pink circle highlights open pyran ring in distal PDT. Reprinted with permission from J. Am. Chem. Soc. 2018, 140, 12808–12818. Copyright 2018 American Chemical Society. (B) Comparison of PDT conformations among Moco found in sulfite oxidase (SUOX), xanthine dehydrogenase (XDH) and DMSO reductase (DMSOR) families. Reprinted with permission from PNAS, 2012, 109 (37) 14773–14778. Copyright 2012 National Academy of Sciences.

Given the global significance of pyranopterin Mo and W enzymes,^3,7,10,14,15^ it is fascinating to note that it is still unclear which features of the PDT ligand play a functional role in the catalytic cycles of pyranopterin Mo and W enzymes.^2,16^ Spectroscopic studies of the PDT component of these enzymes have been limited,^17−26^ and there have been no methods available to directly probe whether any chemical changes occur at the PDT ligand, leaving its function in enzymatic catalysis undetermined. Although numerous pyranopterin Mo and W enzymes have been structurally characterized by X-ray crystallography,^3,10,16,27^ these data have not yielded information that might allow for an interpretation of the role of the PDT ligand in catalysis. Several of the most significant results obtained from protein crystal structures reveal key information regarding potential roles for the PDT. In some enzymes, the PDT ligand is positioned between the Mo/W ion and exogenous redox cofactors that include FeS clusters and flavins (Figure 2A, yellow circle).^16^ This geometric arrangement has been interpreted as evidence that the PDT ligand functions as a conduit for the transfer of electrons between the metal ion and its redox partners.^28^ In several crystal structures of enzymes that belong to the DMSOR family, one of the two PDT ligands does not possess a pyran ring; instead, the pyran ring has been cleaved open (Figure 2A, pink circle).^29−31^ Interestingly, pyran ring opening can lead to a novel thiol-thione dithiolene structure that can dramatically affect the electronic structure and reduction potential of the metal site.^32−34^ Curiously, the ‘open’ PDT is always found to be the ligand that is not involved in H-bonding to FeS redox cofactors. These results have led to suggestions that the ‘open’ form of the PDT ligand serves to modulate the reduction potential of the metal ion using the significantly different electronic environment of an open PDT form of the ligand.^1^ A third observation is that there are notable differences in the PDT conformation among the numerous protein crystal structures with respect to the degree of bending or nonplanarity of the PDT ligand (Figure 2B).^27^ It has been determined that there is a correlation between the degree of PDT conformational distortions and the enzyme family type.^27^ These differences in pterin conformation also correlate with different pterin oxidation states, thus calling into question what role redox reactions of the PDT pterin might play in enzymatic catalysis. Moco is recognized as an extraordinarily redox rich entity, where the Mo ion and the dithiolene are each 2- electron redox active,^2,28,34−40^ and the pterin component of a pyranopterin is also capable of two-electron redox reactions.^2,27,32,33,37,41−45^

The ambiguity surrounding the role(s) of the PDT in Mo and W enzymes results from the difficult challenges of obtaining experimental data that directly probes the PDT component of Moco^19,20^ to reveal changes at the pterin within holoenzymes. While it might be proposed that Moco extracted and isolated from the protein could be studied to provide information about PDT reactivity, this approach removes the multiple hydrogen bonding arrangements that are specific to a given protein. Furthermore, such efforts are hindered by the high instability of the isolated Moco outside of the protein environment.^46−48^ As a result, the above hypotheses regarding the possible functions of the PDT ligand remain unresolved. It is within this context that we embarked some years ago on developing a model system that would permit detailed analyses of both the geometric and electronic structure of a pyranopterin dithiolene, and the chemical behavior of this ligand when bound to a relevant oxo-molybdenum center.^49,50^ Here we report the synthesis and characterization of the only known synthetic reduced pyranopterin dithiolene ligand (Figure 1B) that closely models the basic MoO(PDT) fragment found in Moco. The [Tp*MoO(S_2_H_2_BMOPP)]^1–^ (**1**) complex detailed here is the first molybdoenzyme model to possess all the key features of the PDT, allowing the reactivity of the unique pyranopterin dithiolene ligand to be studied for the first time. The results provide new insights into the nature of the PDT ligand of Moco and reveal how a pyranopterin appended to the dithiolene chelate can modulate reactivity at both the Mo ion and the PDT pterin.

## Results

### Synthesis and Characterization

**Figure 3:**
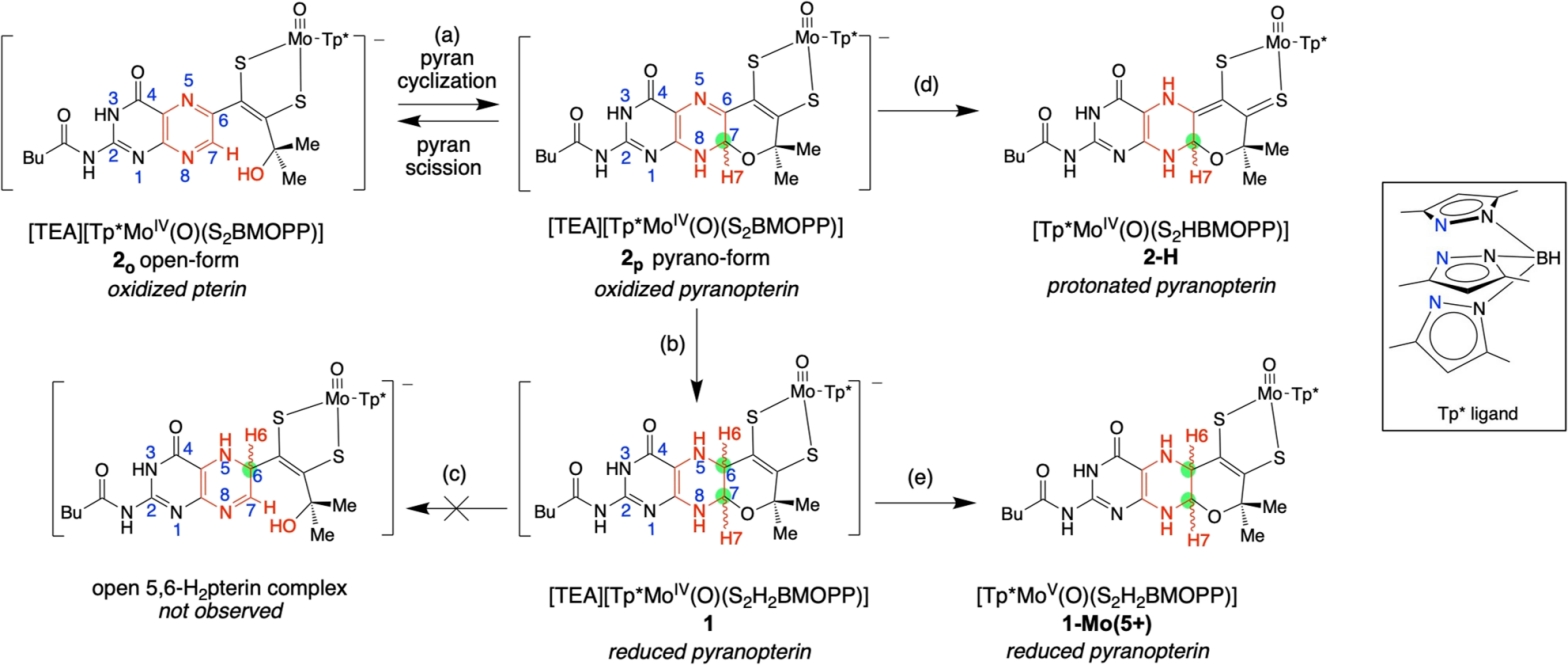
(a) Equilibrium of pyran cyclization in precursor complex **2**. (b) 1 equiv KBH_4_, MeOH, 0.05% H_2_O/ACN, NH_4_Cl, 25 °C. (c) Unobserved pyran ring opening in reduced pyraopterin complex **1**. (d) TFAA, ACN. (e) 1 equiv ferrocenium hexafluorophosphate, ACN. Inset: structure of Tp* = *tris*(3,5-dimethylpyrazolyl)hydroborate; the three blue N atoms form a tridentate ligand on Mo. Green dots highlight chiral C atoms.

Compound **1** is the target molecule from the final step of a multistep synthetic scheme developed over a decade (Scheme S1, step (d)). Reduction of the pterin portion of the precursor (TEA)[Tp*Mo^IV^O(S_2_BMOPP)] (**2**), (TEA = tetraethylammonium; Tp* = *tris*(3,5-dimethylpyrazolyl)hydro-borate; BMOPP = 6-(3-butynyl-2-methyl-2-ol)-2-pivaloyl pterin) is accomplished through reaction with potassium borohydride (Figure 3, (b)) to produce a pale yellow solid in ∼ 70% yield. The known fluxional behavior of the pyran ring in the precursor **2** exists in solution as both the uncyclized open form **2**_**o**_ and the cyclized pyrano form **2**_**p**_, (Figure 3, (a)).^51,52^ Despite the possibilty of forming a mixture of reduced pterin complexes from **2**_**o**_ and **2**_**p**_, our results show that only the reduced pyranopterin complex **1** is formed and no evidence for any other reduced pterin products was obtained.

Isolated **1** is air-sensitive and regenerates **2** by oxidation. Anaerobic solutions of **1** in methanol are stable for several days as monitored by NMR, whereas solutions of **1** in acetonitrile undergo some pivaloyl group cleavage within 24–48 h. Efforts to obtain single crystals suitable for X-ray diffraction analysis were unsuccessful, and this is likely due to the solution instability in addition to the presence of multiple enantiomeric diastereomers (see below). However, the precursor complex **2**, as well as its one-electron oxidized derivative [Tp*Mo^V^O(S_2_BMOPP)] (**2-Mo(5+)**) have both been structurally characterized by X-ray diffraction and crystals of both compounds only exhibit the pyrano-conformer.^51^ The molecularity and pyranopterin conformation of **1** was confirmed by high resolution mass spectrometry (Figure S1) and 2D NMR methods (^1^H, COSY, HSQC, NOESY) (Figures S5–S10), while additional characterization methods include FTIR (Figure S2) and cyclic voltammetry. EPR spectroscopy was used to characterize the one electron oxidized complex of **1**, [Tp*Mo^V^O(S_2_H_2_BMOPP)], **1-Mo(5+)**. Electronic absorption spectroscopy was used to monitor oxidation reactions of **1**, including identification of the oxidation products.

### NMR Characterization of Complexes

There are three stereocenters in **1** and these are located at the Mo atom, and at positions C6 and C7 of the pyranopterin dithiolene ligand (Figure 3, green dots on **1**). The two stereocenters on the pterin create the possibility of four possible diastereomers for each enantiomer of **1** with configurations at positions C6 and C7 of *R,R-, S,S-, R,S-,* and *S,R-*. Of these four isomers, NMR results are only consistent with the presence of the *cis-R,R* and *cis*-*S,S* diastereomers in a 1:1 ratio as determined by both COSY and NOESY spectra (Figures S6–S10). Density functional theory (DFT) computations^53^ confirm that the *R,R-* and *S,S-* diastereomers are the lowest energy diastereomers with the *R,R-* configuration being slightly (1.4 kJ/mol) lower in energy (Figure S3). The *R,S-* and *S,R-* isomers are significantly higher in energy (12.1 and 20.4 kJ/mol, respectively) when compared to the computed *R,R-* structure (Figure S3). A similar situation was found previously for **2-H** where the two diastereomers of *R-* and *S-* chirality at C7 (Figure 3, green dot on **2-H**) are close in energy.^32^ Note that each of the diastereomers are additionally chiral at the Mo atom, where the enantiomers can be distinguished according to the relative direction of the pterin dithiolene ligand, left or right, when the Mo≡O group is oriented along the + *z* axis. It has been noted that the orientation of the PDT relative to the Mo≡O group is different in SO and XDH family enzymes.^54^ The bond line drawing of **1** shown in Figure 3 depicts the pyranopterin dithiolene ligand positioned to the left relative to the vertically aligned Mo≡O axis vector, and this is the orientation found in XDH family enzymes. The absence of any molecular symmetry in either of the *R,R-, S,S-* diastereomers means every proton and methyl group is unique. NOESY spectra (Figures S5–S10) interpreted in conjunction with the DFT-optimized structures (Figure S3) allowed assignment of every proton in both diastereomers, as shown for selected NMR regions in Figure S5, thereby providing confirmation of the structure of **1**. In addition, the pterin proton assignments in Figure S5 for the most stable *R,R-* diastereomer correspond well with those reported for the *R,R-* reduced pyranopterin in Precursor Z (Figure S11), the biochemical precursor to the PDT, where *R-*H7 at 5.26 ppm and *R-*H6 at 3.7 ppm (J 1.8 Hz) in **1** compares well with 5.39 and 3.62 ppm (J 1.7 Hz) for the same protons in Precursor Z.^55^

Given the examples of a pyran cleaved PDT in several enzymes,^29−31^ we were curious whether the reduced pyranopterin in **1** would exhibit reversible pyran ring cleavage/cyclization at the C7–O bond in a manner similar to that of **2** (Figure 3).^52^ Surprisingly, the ^1^H NMR spectrum shows the presence of only the reduced pyranopterin conformation of **1** and provides no evidence for pyran ring cleavage that derives from a ring chain tautomerism process,^56^ which is indicated by a downfield resonance below 9 ppm for H7. If pyran cleavage did occur in **1**, it would access an unstable 5,6-dihydropterin form. A ring-open form of **1** was computed and the optimized structure is 24.5 kcal/mol higher in energy than that of *R,R-***1**. In view of these experimental and computational results, we conclude that pyran ring cleavage is energetically unfavorable for ring-closed reduced PDTs.

### Impact of Pterin Oxidation State and Conformation

**Figure 4:**
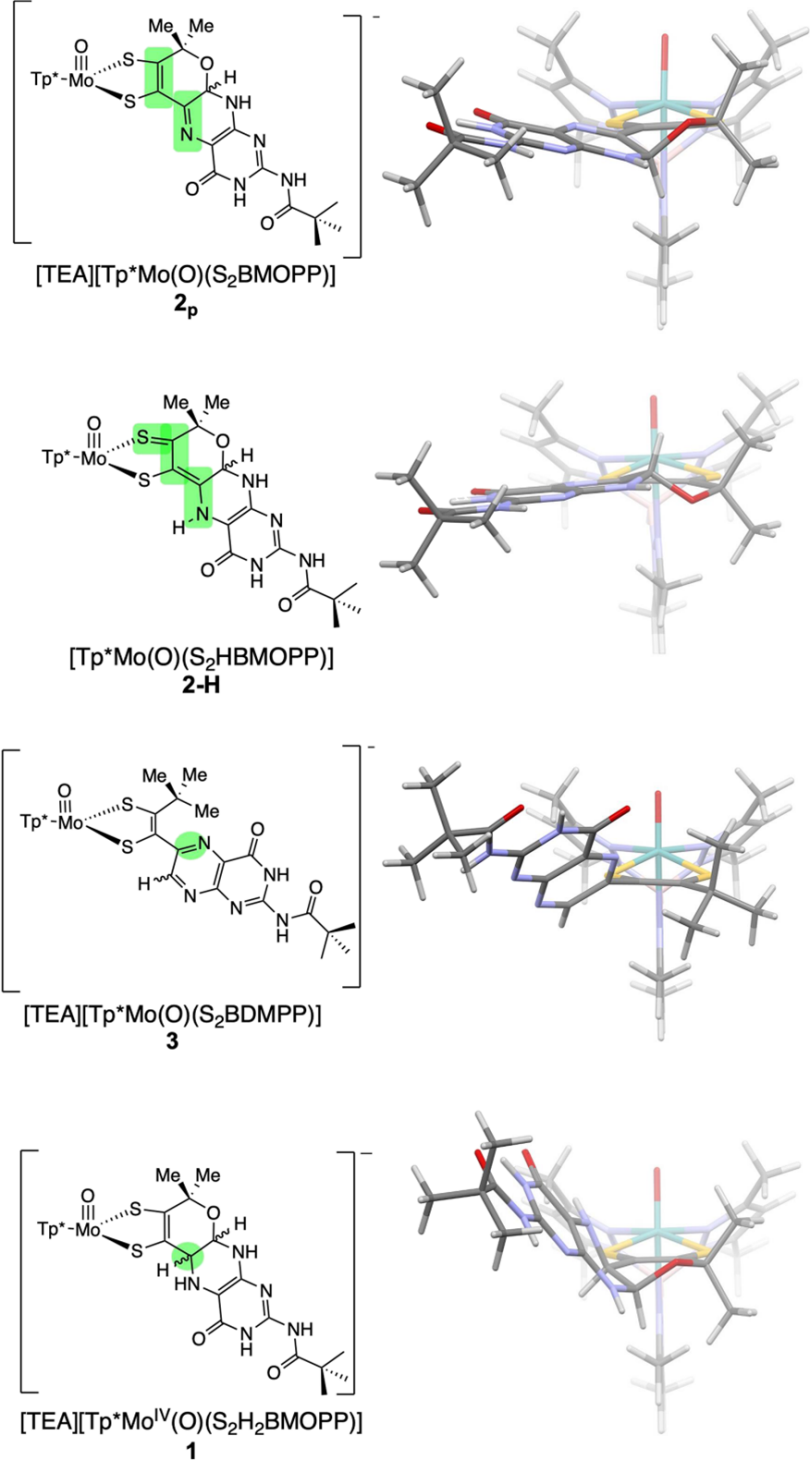
Pyranopterin conformation determines its electronic influence on dithiolene component of the PDT. The nearly coplanar pterin and dithiolene in **2**_**p**_ allows conjugation between dithiolene and pterin as highlighted in green in the bond line drawing. The saturated C6 (green circle) in *R*,*R*-**1** results in a severely distorted pterin conformation relative to the dithiolene, and this prevents conjugation. The pterin in **2-H** possesses thiol-thione resonance character (green highlight). The pterin in **3** is rotated out of planarity with respect to the dithiolene due to the lack of a pyran ring and steric interactions that combine to decrease conjugation between the N5–C6 bond (green highlight) and the dithiolene.

One major objective of this project has been to determine the impact of the pterin substituent on the dithiolene chelate and how this interaction affects Mo reactivity. We now have synthetic examples of oxo-Mo dithiolene complexes substituted by pterins in different oxidation states, conformations and protonation states. This allows for a comparison of data that report on the electronic environment of the Mo ion, indicating precisely how the nature of the pterin impacts the dithiolene chelate and the Mo ion. A combination of infrared and electronic absorption spectroscopies, coupled with electrochemistry studies for the four complexes illustrated in Figure 4 are collected in Table 1. Table 1 also includes the Δ(C–S) parameter, which is defined as the difference in the two C–S bond distances in the dithiolene chelate. This parameter is a measure of the asymmetry in the dithiolene ligand, which in turn conveys the extent of thione/thiolate resonance character admixed into the electronic ground state leading to partial dithiolene oxidation.^1,32^ For each of the four model complexes **2**_**p**_, **2-H**, **3,** and **1** in Figure 4, a bond line drawing at the left depicts differences in the pterin group, and the structural view on the right highlights the pterin conformation relative to the dithiolene ligand. In this figure, all four molecules have the same orientation with the Mo≡O group aligned along a vertical *z*-axis and the Tp* ligand greyed out in the back. We previously reported in this *Journal* the use of complexes **2**_**p**_ and **3** to investigate how the dithiolene electronic structure differs significantly for a pyranopterin versus an uncyclized, ring-opened pyranopterin.^8,9^ In a subsequent publication, we showed how pterin protonation in **2-H** (Figure 3, (**d**)) leads to striking changes at the Mo ion, notably the large +300 mV shift in the Mo(5+/4+) reduction potential.^32^ In both reports, dithiolene asymmetry was observed and attributed to a thione/thiolate resonance structure contribution to the electronic ground state that is accessed when the pyranopterin and dithiolene moieties are coplanar. The acquisition of the reduced pyranopterin complex **1** reported here adds the complementary member of the compound set shown in Figure 4, which now includes examples of all three pterin oxidation states (**3** - oxidized, **2**_**p**_ and **2-H** - dihydro, and **1** - tetrahydro) and examples of ring-open versus ring-closed pyranopterin conformations. Perusal of the data in Table 1 reveals that the electronic environment at the Mo atom is unexpectedly most similar for complex **1**, which possesses a reduced pterin, and complex **3** that incorporates an oxidized (open) pterin. In contrast, the electronic impact of the pterin in **1** and **3** significantly differs from that produced by the semireduced pterins in **2** and **2-H**. The Δ(C–S) parameter reveals the degree of asymmetry in the dithiolene that underlies the observed electronic differences in these model complexes. Thus, the dithiolene chelate in **1** and **3** shows little asymmetry and is best described as an ene-dithiolate donor, whereas the chelates in **2** and **2-H** have substantial asymmetry, especially in **2-H**, that is characteristic of the ligand possessing increased thione/thiolate character. It is the poorer π-donor ability of the partially oxidized thione/thiolate chelate in **2** and **2-H** vs the better π-donor ene-dithiolate ligand in **1** and **3** that accounts for the observed differences in Mo≡O vibrational stretching frequencies and in the Mo(5+/4+) reduction potentials. The dominant ene-dithiolate character in **1** and **3** is consistent with the chelate being electronically insulated from the pterin. The saturated bridgehead carbon C6 in **1** interrupts communication between the dithiolene and pterin. The pterin is rotated out of planarity with the dithiolene in complex **3**, decreasing the π-conjugation between the dithiolene and the pterin and dramatically reducing any electronic influence by the pterin on the dithiolene. We note that the formalism developed by Enemark to identify the total number of electrons (n) associated with a particular redox state of Moco,^57^ can be applied as follows. Compound **1** contains the [Moco]^8^ unit, **1-Mo(5+)** contains the [Moco]^7^ unit, whereas **2–H-Mo(5+)** and **2-H** exhibit alternative electron distributions within the [Moco]^6^ unit.

**Table 1:** Comparison of Spectroscopic Data and Reduction Potential for **1**, **2**, **2-H**, and **3**

complex	ν (Mo=O) cm^–1^	*E*_(Mo5+/4+)_V (vs Fc^+^/Fc)	λ, nm (ε, M^–1^cm^–1^)	Δ(C–S), Å^a^
**2_p_**	924	–520	450 (14,500)	0.03
			375 (14,000)	
**2-H**	938	–174	526 (27,800)	0.06
**3**	916	–574	337 (8,950)	0.014
**1**	918	–570	375 (10,100)	0.005^b^
			445 (7,020)	

a Δ(C–S) reports the difference in C–S bond distances in dithiolene.

b From computed structure for *R,R-***1.**

### EPR Spectroscopy

**Figure 5:**
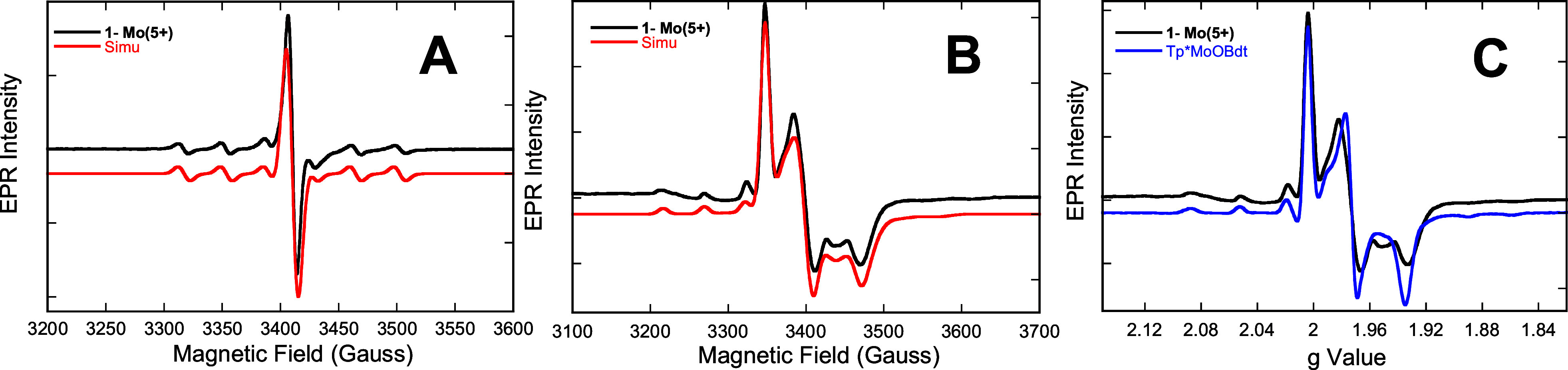
(A) Room temperature EPR spectrum and simulation of **1-Mo(5+)** in acetonitrile. (B) 77K EPR spectrum and simulation of **1-Mo(5+)** in an *n*-butyronitrile glass. (C) Overlay of 77K EPR spectra of **1-Mo(5+)** and model compound Tp*Mo^(V)^O(bdt) (in toluene glass). Note that even though Tp*Mo^(V)^O(bdt) possesses no pyranopterin ligand, the EPR spectra in C are very similar, suggesting that EPR is not sensitive to pyranopterin electronic changes.

The above discussion establishes that the nature of the pterin component of the PDT can have a measurable, and in some cases a quite large, impact on the electronic structure of these complexes and, by extension, catalytic processes that occur in the enzymes. EPR spectroscopy has been heavily employed to probe the geometric and electronic structure of both synthetic models and enzyme active sites that are in the paramagnetic d^1^ Mo(V) state.^58−61^ Here, we have used EPR spectroscopy to better understand how the pterin component of the PDT influences the electronic structure of the Mo ion in the one-electron oxidized species, **1-Mo(5+)**. The **1-Mo(5+)** complex was generated *in situ* by adding 1 equiv of the 1e^–^ oxidant ferrocenium hexafluorophosphate, and the EPR signal of **1-Mo(5+)** appears instantly upon addition of the oxidant. The room-temperature and 77K EPR spectra of **1-Mo(5+)**, as well as their spectral simulations, are displayed in Figure 5A and 5B. The **1-Mo(5+)** spin Hamiltonian parameters determined from these spectral simulations are listed in Table S1 and are the first determined for a reduced pyranopterin dithiolene oxo-molybdenum complex. The spin Hamiltonian parameters for **2-Mo(5+)**, **3-Mo(5+)**, and the model complex Tp*MoO(bdt) (bdt = 1,2-benzenedithiolate) are also tabulated for comparison purposes. Consideration of these data shows that the spin-Hamiltonian parameters among this set of Mo(V) species exhibit very little variation in their g- and A-tensor values as the pterin changes oxidation state or structure. In fact, the data are very similar to the parameters previously obtained for Tp*MoO(bdt),^32,62^ which does not possess a pterin substituent or a pyran ring (Figure 5C). It is astounding to discover that the EPR spectra do not strongly reflect the oxidation state and conformation of the pterin ring that is appended to the dithiolene. These data support a conclusion that the degree of Mo-ligand covalency, the nature of the ligand field splitting, spin–orbit coupling, and the mixing of charge-transfer excited states into the electronic ground state are highly similar for these complexes.^63−70^ EPR spectroscopy is expected to reveal changes at the pterin in Mo or W enzymes when the PDT possesses a large degree of covalent thiol-thione resonance character (Figure 4)^32−34,71,72^ admixed into the electronic ground state at the Mo(V) level (e.g.**2–H-Mo(5+)**). However, the X-ray structure of **2-Mo(5+)**, coupled with computed optimized structures for **1-Mo(5+)** and **3-Mo(5+)**, show small values for the Δ(C–S) parameter and this indicates all these Mo(5+) complexes possess very little thione/thiolate resonance character. The small values for Δ(C–S) and the associated experimental and computed EPR spin-Hamiltonian parameters (Table S1) are all consistent with a dithiolate form of the pterin dithiolene ligands in (**1–3)-Mo(5+)**. As such, EPR spectroscopy is not likely to be sensitive to changes at the pterin in Mo or W enzymes at the 5+ oxidation state when the PDT has dominant dithiolate character. Furthermore, our results suggest the partially oxidized thione/thiolate is likely to be found only in the Mo(4+) species.

### Electrochemical Characterization

**Figure 6:**
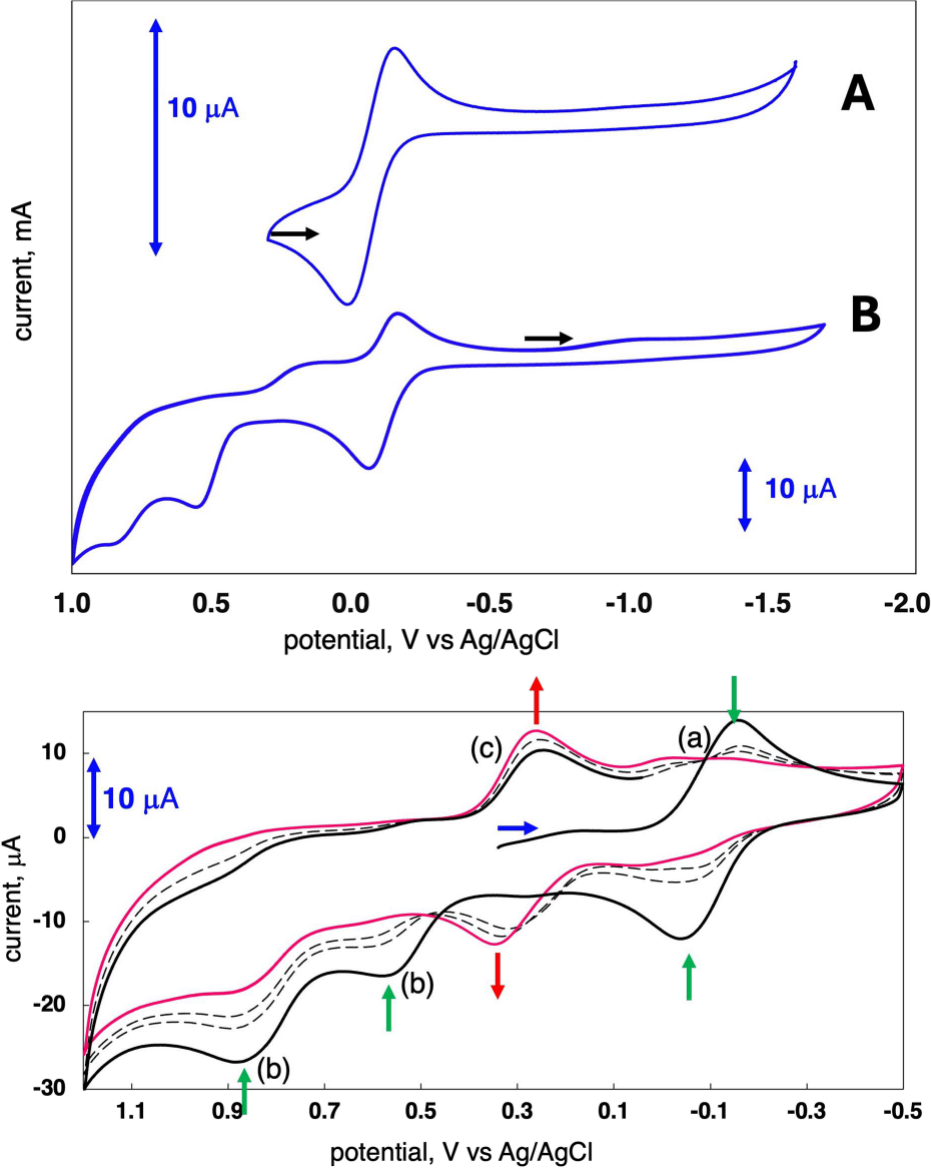
Cyclic voltammograms of **1** plotted vs the AgCl/Ag reference electrode in ACN. (top) Voltammograms obtained at a scan rate of 100 mV/s. Black arrows indicate initial potentials. (A) The Mo(5+)/(4+) couple is the only feature observed when the potential range is limited to +300–1600 mV. (B) Increasing the positive potential window to +1 V reveals additional redox processes. (bottom) Cycling between +1200 and −500 mV at 2 V/s reveals decay and growth of species. Blue arrow indicates initial potential. The black line is the initial scan, the red line is the final scan, and only two of 14 cycles are shown as dashed lines for clarity. Note sample concentrations differ for scans at top and bottom, and this is indicated by the blue arrow showing a 10 μA interval.

Cyclic voltammetry was used to investigate the redox behavior of **1** in acetonitrile. Figure 6 (top) shows two voltammograms obtained at a slow scan rate of 100 mV/s. A single reversible couple at −130 mV vs the AgCl/Ag reference electrode (−570 mV vs Fc^+/0^) in the +250 to −1600 mV potential range is assigned to the Mo(5+/4+) one-electron redox process (Figure 6, top, **A**), while enlarging the potential window reveals two irreversible oxidations and a new irreversible reduction wave (Figure 6, top, **B**). Additional information about these irreversible processes is obtained by increasing the scan rate to a fast 2 V/sec (Figure 6, bottom). The Mo(5+/4+) couple (a) decays in parallel with decay of the irreversible processes (b) near +550 and +900 mV (green arrows), while simultaneously a new reversible couple (c) appears at ∼+300 mV (red arrows). It appears that the Mo(5+/4+) couple initially at −130 mV (vs AgCl/Ag) shifts positive to ∼ + 300 mV. In fact, the couple at +240 mV (−200 mV vs Fc^+/0^) in Figure 6 (bottom) can be assigned to the protonated complex **2-H** that we have previously reported exhibits a Mo(5+/4+) potential at −205 mV vs Fc^+/0^.^32^

**Scheme 1:**
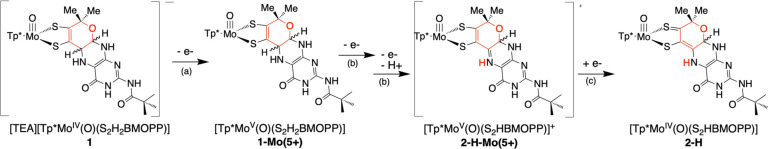
Proposed Sequence of Redox Events during Cyclic Voltammetry at 2 V/s Redox steps (a), (b), and (c) correspond to processes labeled in Figure 6 (bottom).

Scheme 1 depicts the proposed interpretation of redox events during rapid cycling (2 V/sec) shown in Figure 6 (bottom). Setting the initial potential at +340 mV generates **1-Mo(5+)** at the electrode surface at the start of the experiment, and this species undergoes a one-electron reduction at −130 mV to Mo(4+) forming **1**, the first species shown in Scheme 1. Reversing the scan direction at −500 mV reoxidizes **1** to **1-Mo(5+)** in step (a). Anodic reactions at +550 and +900 mV in step (b) are two one-electron oxidations at the pterin, converting the fully reduced pterin to a protonated dihydropterin compound, **2–H-Mo(5+)**, following loss of a proton. Reversing the direction again at +1200 mV allows observation of **2–H-Mo(5+)** reduction to **2-H** near +240 mV in step (c). Species **2–H-Mo(5+)** is unstable and its reduction by 1 electron to **2-H** is only observed at fast (>1 V/sec) scan rates. Consistent with this assignment for step (c) is our previous report on the redox behavior and full characterization of **2-H**.^32^ In addition, we have been unable to spectroscopically observe the protonation of **2-Mo(5+)** to **2–H-Mo(5+),** confirming the instability of the proposed **2–H-Mo(5+)** species.

### Oxidation Reactions

**Figure 7:**
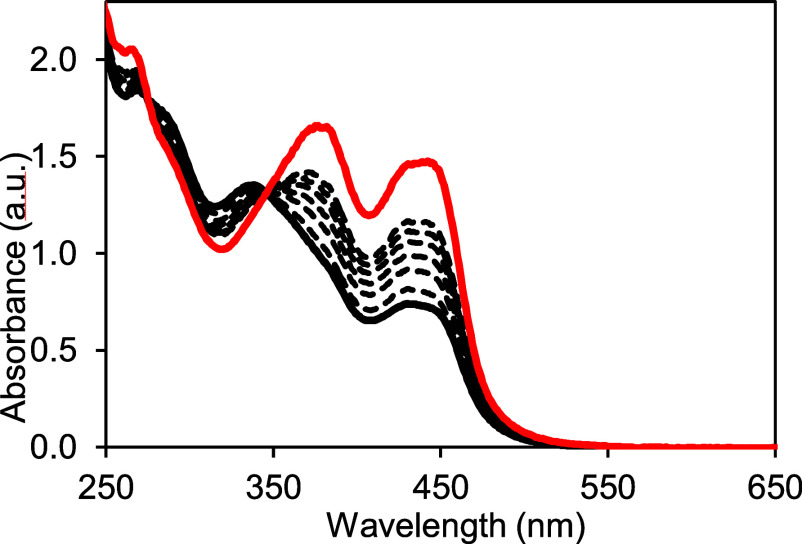
Air oxidation of **1** in acetonitrile. Black line is initial spectrum of **1**; dashed lines show changes due to oxidation at 1, 5, 10, 20, 25, and 90 min. Red line corresponds to **2** where the reduced tetrahydro-pterin has been oxidized to dihydropterin by 2e^–^/2H^+^. The best fit to the time dependence at 448 nm using an exponential rise function gave a rise time of 3.34 × 10^–2^ min^–1^ (Figure S20).

The redox reactivity of **1** was explored in air, in addition to using the 1- and 2-electron oxidants ferrocenium (Fc^+^) and dichlorophenolindophenol (DCIP). These experiments were performed in both protic (MeOH) and aprotic (ACN) solvents, monitored by electronic absorption and EPR spectroscopies, and in some cases by ESI-MS. Given the well-known fragility of Moco when removed from its protein surroundings, it is not surprising to find that **1** is unstable toward air. Monitoring the electronic absorption spectrum of an acetonitrile solution of **1** (Figure 7) shows the growth of new spectroscopic features, notably absorbances at 380 and 440 nm, that are characteristic of the pyranopterin complex **2**.^8^ The isosbestic point at 344 nm indicates that air oxidation of **1** initially occurs only at the reduced pterin moiety to regenerate the semireduced pyranopterin structure. Subsequent oxidation of **2** under aerobic conditions is known to result in Mo oxidation to form **2-Mo(5+)** (Figure S12, in MeOH). Since the pyranopterin group remains intact without pyran ring cleavage during air oxidation, these observations underscore the robustness of the pyranopterin structure, which may have a functional role in preventing general deterioration of the dithiolene ligand.^43^

### Oxidation Reactions with DCIP

**Figure 8:**
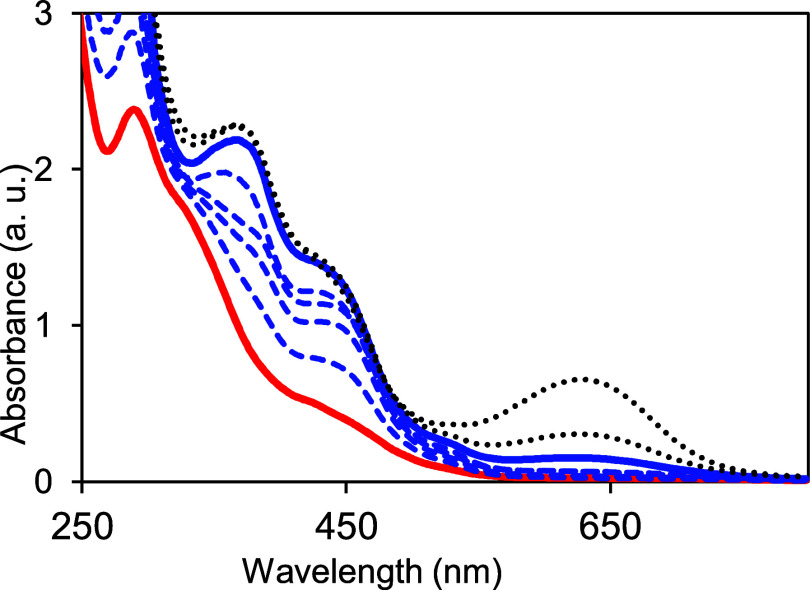
Oxidation of **1** as aliquots of DCIP are added in methanol. Red line is **1** without DCIP; blue dashed lines are 0.25, 0.4, 0.5, and 0.75 equiv DCIP, blue line is 1.0 equiv DCIP, and black dotted lines are 1.1 and 1.25 equiv DCIP. Samples were allowed to equilibrate for 19 h before measuring.

DCIP is a common redox reagent in assays, and was used as an oxidizing agent to probe the redox reactivity of Moco over four decades ago when the pyran ring of the PDT was an unknown structural component of Moco.^11,12^ We have previously demonstrated that a reduced pyranopterin molecule can be oxidized in a 2e^–^/2H^+^ reaction that leads to pyran ring opening and a fully oxidized pterin.^43^ The availability of model complex **1**, which possesses both the reduced pyranopterin as well as the dithiolene component of Moco, allows us to establish how an appended dithiolene affects the pyranopterin oxidation process. Figure 8 shows the titration of a methanol solution of **1** with DCIP as monitored by electronic absorption spectroscopy. The intense blue color of oxidized DCIP derives from the strong absorption at 650 nm, and this absorption feature is absent following addition of 0.25–1.0 equiv DCIP to **1** due to the reduction of DCIP to colorless H_2_DCIP. Spectra recorded for this aliquot range exhibit the growth of absorption maxima at 380 and 440 nm that are characteristic of **2**. The absorption at 650 nm reappears when the DCIP addition exceeds 1 eq, signaling the termination of the redox reaction. Pterin oxidation by DCIP is much slower than air oxidation: substoichiometric samples required almost 20 h for complete bleaching of the DCIP 650 nm absorption. When the DCIP oxidation of **1** is performed in aprotic acetonitrile solvent, the reaction proceeds extremely slowly such that DCIP bleaching is not accomplished even after 48 h. These observations are interpreted as resulting from the proton dependent nature of DCIP redox reactivity such that the reaction can proceed, albeit slowly, in protic methanol but not in aprotic acetonitrile. Use of EPR to monitor the DCIP oxidation of **1** shows only a very small signal from **1-Mo(5+)**, indicating negligible oxidation of the Mo(4+) ion (Figure S13). This result confirms that oxidation by DCIP only occurs at the pterin and not at the Mo ion. This observation is consistent with our previous studies demonstrating a lack of Mo(4+) oxidation in **2** by DCIP.^11^ Only when the pyranopterin is protonated, such as in **2-H**, will DCIP oxidize Mo(4+) by one electron to the Mo(5+) state.

### Oxidation Reactions with Ferrocenium

**Figure 9:**
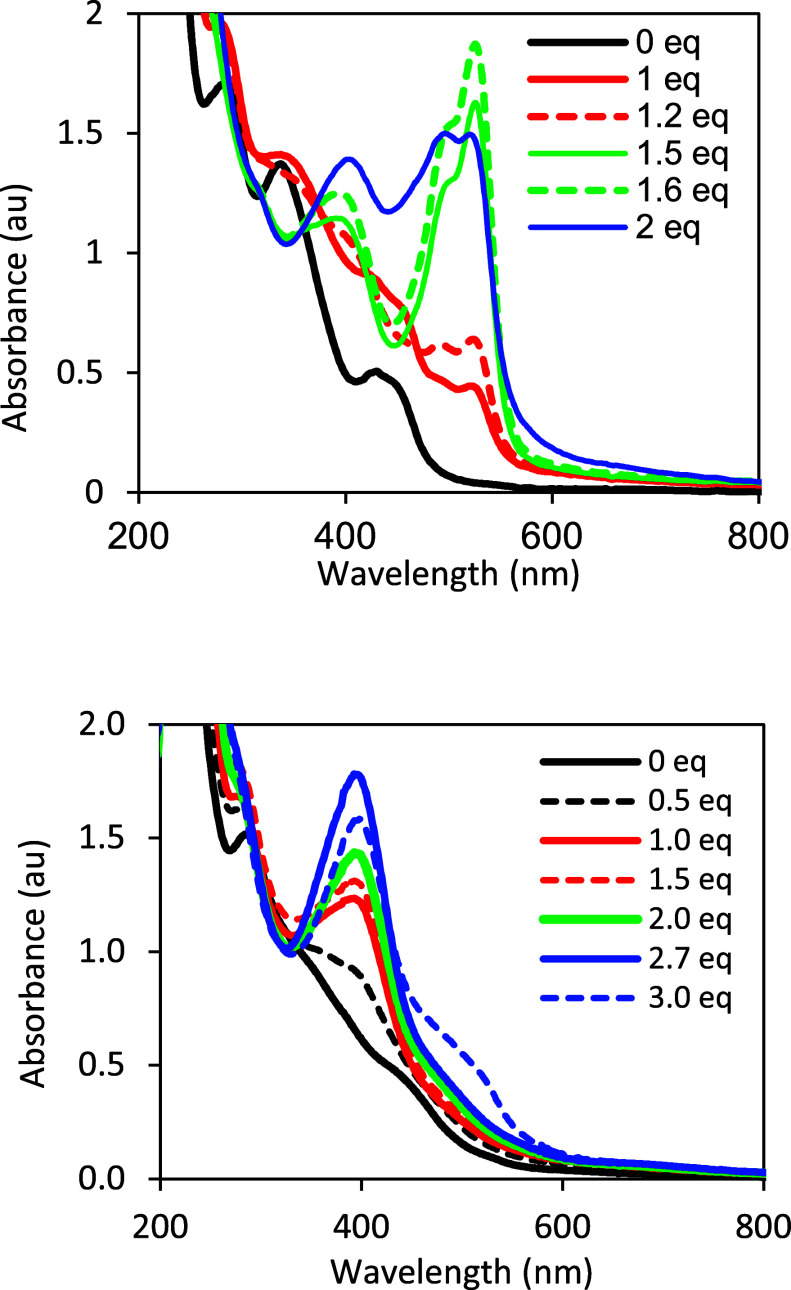
Titration of **1** with >1 equiv Fc^+^. (Top) In acetonitrile, 1 equiv Fc^+^ oxidizes **1** (black line) to **1-Mo(5+)** (red line). Addition of 1.5 and 1.6 equiv Fc^+^ causes the growth of a species absorbing at 526 nm (green lines). This species is the previously reported protonated pyranopterin complex [Tp*Mo^IV^O(S_2_HBMOPP)] (**2-H**) based on the intense absorption at 526 nm that is the characteristic spectroscopic signature of **2-H** (Figure S18). Addition of 2 equiv Fc^+^ causes the decay of the 526 nm absorption of **2-H** and the growth of absorptions at 400 and 490 nm (blue line). (Bottom) In methanol, Fc^+^ aliquots (0.5 to 3 equiv) cause a new absorption at ∼ 400 nm that is assigned to **2-Mo(5+)**; no formation of the protonated pterin complex [Tp*Mo^IV^O(S_2_HBMOPP)] **2-H** is observed until 3 equiv Fc^+^ are added, at which time a small absorption near 500 nm appears.

Ferrocenium (Fc^+^) hexafluorophosphate is a convenient 1-electron oxidant for nonaqueous systems. The reaction of Fc^+^ with **1** has very different outcomes than the air and DCIP oxidations described above. Titration of **1** in ACN with 0.2–3.5 equiv aliquots of Fc^+^ monitored by electronic absorption spectroscopy results in a complicated series of spectral changes indicating several product species (Figure S14). The absorption spectra in Figure 9, (top) compares the initial spectrum of **1** (black line) and the 1-electron oxidized product [Tp*Mo^V^O(S_2_H_2_BMOPP)], **1-Mo(5+)** (red line) after addition of 1 eq Fc^+^. The ferrocenium oxidation of **1** occurs within seconds and monitoring the reaction solution shows no further changes in the absorption and EPR spectra over 24 h (Figures S15 and S16). The electronic absorption spectral changes show that the absorption bands shift to lower energy as the d^2^ Mo(4+) ion is oxidized to the d^1^ Mo(5+) ion. This is consistent with the lowest lying d(xy) redox orbital being half filled in the Mo(5+) state allowing for low energy dithiolene → Mo(xy) ligand-to-metal charge transfer transitions.^28,58,70,73,74^ The appearance of a characteristic Mo(5+) EPR signal (Figure S15, bottom) confirms a simple one-electron oxidation of Mo(4+) in **1** to **1-Mo(5+)**. This interpretation is also confirmed by ESI-MS data (Figure S17).

Addition of >1 equiv Fc^+^ to **1** results in further oxidation where the outcomes depend on the nature of the solvent: results obtained in aprotic acetonitrile and in protic methanol are compared in Figure 9. Addition Fc^+^ in excess of 1 equiv causes the formation of the previously reported protonated pyranopterin complex [Tp*Mo^IV^O(S_2_HBMOPP)] (**2-H**)^32^ based on the intense absorption at 526 nm, which decays as the Fc^+^ titration proceeds. In methanol (Figure 9, bottom), Fc^+^ titration shows the initial formation of **1-Mo(5+),** then subsequent oxidation to **2-Mo(5+)**. No formation of the protonated pterin complex [Tp*Mo^IV^O(S_2_HBMOPP)] **2-H** is observed.

**Figure 10:**
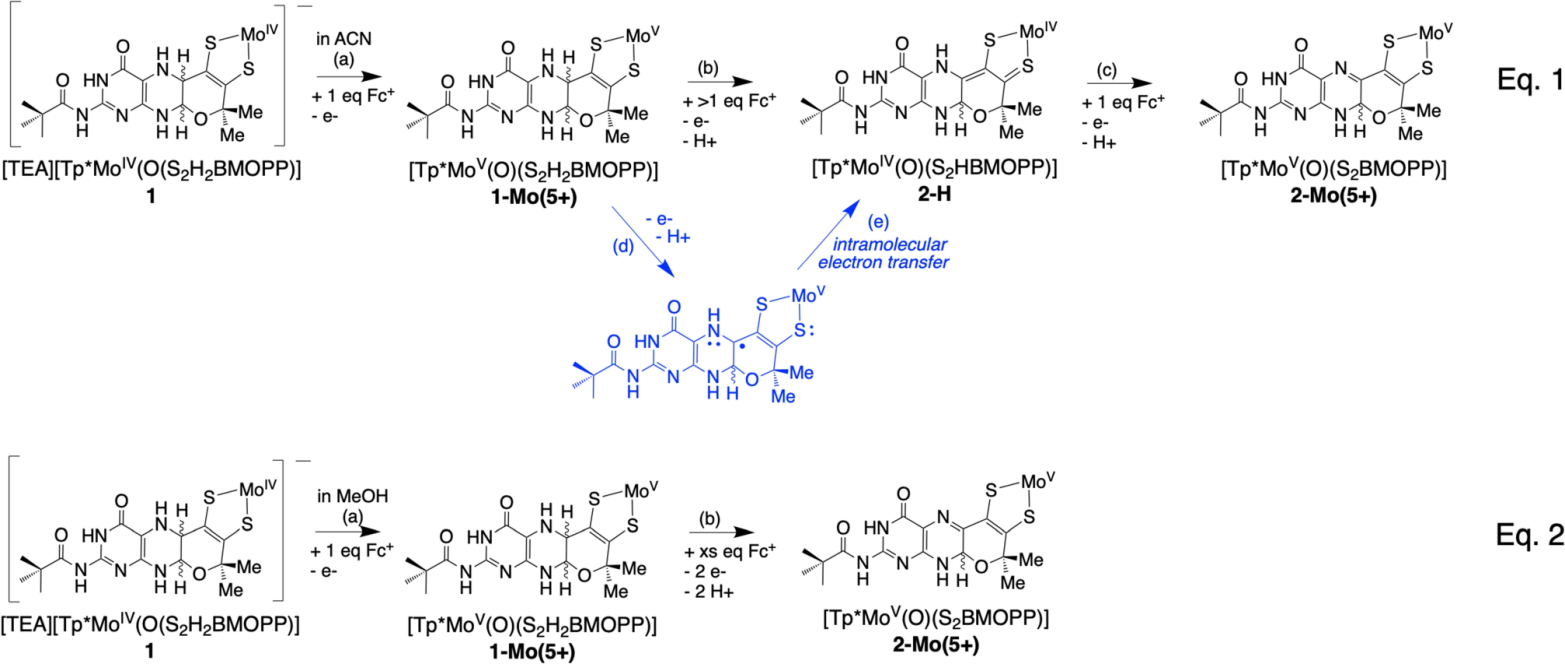
Equation 1 shows the outcome of Fc^+^ addition to **1** in ACN while eq 2 depicts the outcome of Fc^+^ addition to **1** in MeOH. For both eqs 1 and 2, the black structures are spectroscopically observed species, and the blue structure (eq 1) is a proposed transient radical species.

**Figure 11:**
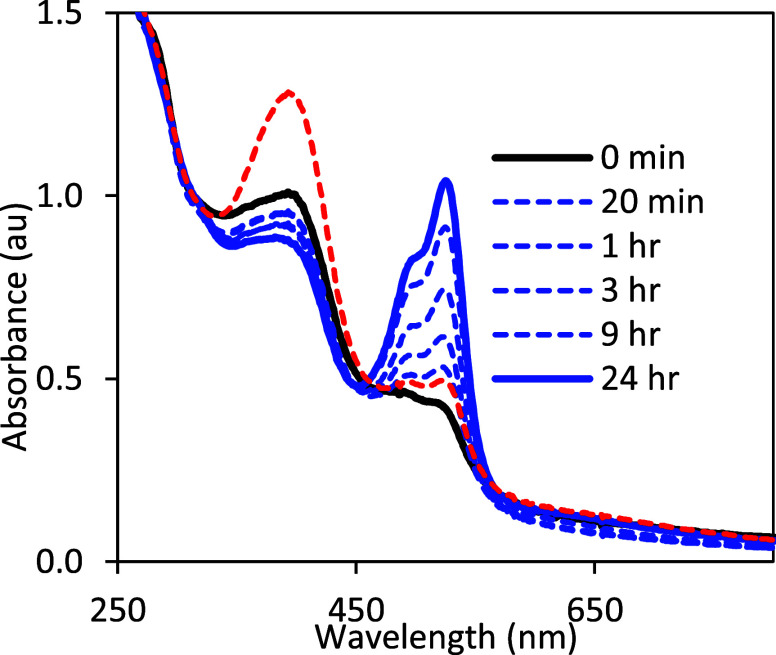
Spectroscopic changes over 0–24 h after 1.4 equiv Fc^+^ added to **1**. Immediately after mixing (black line, *t* = 0 min) absorptions at 400 and 520 nm indicate a mixture of **1-Mo(5+)** and a very small amount (<4%) of **2-H**. During a 24 h period, the absorption at 526 nm increases to a maximum of 1.03 au (blue line).

The results in Figure 9 clearly show how the nature of the solvent affects Fc^+^ oxidation of **1**. Figure 10 summarizes these oxidation outcomes in ACN and MeOH in eqs 1 and 2. In particular, the formation of the Mo(4+) complex **2-H** resulting from the addition of Fc^+^ oxidant to **1-Mo(5+)** in acetonitrile (eq 1) was both unexpected and interesting. In contrast to the initial oxidation of **1** to **1-Mo(5+)** that is complete within a minute, the absorption at 526 nm corresponding to **2-H** grows in slowly over 24 h. Figure 11 shows the series of spectra recorded after 1.4 equiv Fc^+^ was added to **1**, which represents a 40% excess of Fc^+^ oxidant. During a 24 h period, the absorption of **2-H** at 526 nm increases to a maximum of 1.03 au. Using the known extinction coefficient for **2-H** (ε_526_ = 27,800 M^–1^cm^–1^), the concentration of **2-H** formed is 0.037 mM, or ∼ 40%, corresponding exactly to the amount of excess Fc^+^. If the cuvette sample is allowed to sit in air for 2 days, **2-H** is oxidized to **2-Mo(5+)** (red dashed line). Repeating this experiment using 1.2 equiv Fc^+^ (20% excess) gives the same results: 20% **2-H** is formed over 24 h (Figure S19).

The formation of **2-H** during Fc^+^ oxidation of **1-Mo(5+)** in acetonitrile represents the 1e^–^ reduction of Mo(5+) back to Mo(4+) concomitant with the 2e^–^/2H^+^ oxidation of the reduced pyranopterin (i.e., a net one-electron oxidation). We propose that the 1e^–^ oxidation of **1-Mo(5+)** occurs at the reduced pterin to generate an unstable pterin radical (Figure 10, (d)) that triggers an intramolecular electron transfer from the pterin radical to Mo (Figure 10, (e)), such that the net reaction generates a 2e^–^ oxidized pyranopterin and the reduced Mo(4+) ion. It is worth emphasizing that the surprising result of Mo(4+) formation as **2-H** from oxidation of **1-Mo(5+)** in acetonitrile is observed in two different experiments within this report, both in the electrochemical investigation of redox processes in **1**, as well as in the chemical oxidation of **1** by ferrocenium. Although pterin radical formation is observed in other metalloenzymes, only a single report in an EPR study of oxidized bacterial aldehyde dehydrogenases provides support for a PDT pterin-based radical in molybdoenzymes.^75^

## Discussion

A reduced pyranopterin dithiolene oxo-molybdenum(IV) complex, **1**, that closely models the molybdenum cofactor (Moco) present in all pyranopterin molybdenum enzymes, has been synthesized for the first time. The importance of this model complex is that it possesses all the key structural features of the MoO(PDT) component of Moco and provides us with an opportunity to study the reactivity and spectroscopy of the unique pyranopterin dithiolene ligand when bound to Mo.

Two main questions were addressed following the successful synthesis and isolation of model **1**. The first question concerned whether a reversible pyran ring cleavage occurred for the reduced pyranopterin portion of the PDT. Such reversible behavior was previously reported for complex **2**, the precursor of **1**, where the oxidized pyranopterin dithiolene ligand exists in solution as an equilibrium of open and closed pyran ring structures (**2**_**o**_ and **2**_**p**_ in Figure 3). Pyran ring opening in pyranopterin Mo enzymes has been proposed as a possible mechanism for how PDT ligands could adjust the reactivity of the Mo ion.^76,77^ Significant evidence in favor of such a process was obtained with the X-ray structure of the bis-PDT cofactor in *E. coli* dissimilatory nitrate reductase (NR), which showed one cyclized pyranopterin and one uncyclized, i.e. ‘open’, pterin in the cofactor.^30^ Contrary to expectation, we have not obtained any evidence for pyran ring opening in **1**. This is important since it suggests that either the open form of **1** represents a higher energy configuration that is less stable than pyran ring-closed **1**, or there is a substantial kinetic barrier to pyran ring opening when the pterin component of the PDT is reduced. Alternatively, if pyran ring cleavage does occur, the molecule undergoes rapid cyclization reforming the pyranopterin such that the open dihydropterin is undetectable. This should not be surprising since the tautomer formed immediately after pyran cleavage is expected to be the 5,6-dihydropterin that is known to be highly unstable. Additionally, the bent and puckered conformation of the reduced pyranopterin may favor cyclization to the pyrano- structure. Extrapolating from this result suggests that the open, uncyclized PDT ligand observed for NR in Nap is more likely from an oxidized, possibly degraded, form of the PDT rather than the reduced pyranopterin form.^30^

The second objective of this study was to establish the nature of the redox reactivity of **1**. As the first available molecule possessing a reduced pyranopterin dithiolene ligand coordinated to Mo in a biologically relevant oxidation state, it was now finally possible to determine whether pterin-based redox could occur.^41,78^ From the results reported here, we have established that the pyranopterin dithiolene ligand imparts a unique reactivity such that **1** undergoes both 1- and 2-electron oxidations, and that oxidation can occur either at the Mo ion or at the pterin group depending on the nature of the oxidant. We observe that air oxidation of the reduced pyranopterin occurs while the ligand remains coordinated to Mo(4+). Proton availability has a strong effect on oxidation outcomes, as illustrated by the proton dependent DCIP reaction where a 2e^–^/2H^+^ oxidation occurs at the pterin in methanol whereas no reaction occurs in aprotic acetonitrile unless the pterin is protonated. In contrast, the one electron oxidant ferrocenium reacts much differently, where the first equivalent of Fc^+^ oxidizes the Mo(4+) ion in **1** to Mo(5+) and the second equivalent initiates oxidation at the reduced pyranopterin group. This pterin oxidation occurs in either ACN or MeOH, but the solvent determines whether intermediate species are observed. In methanol, ferrocenium causes a net 3e^–^ oxidation reaction where both the Mo and the pterin are oxidized by 1- and 2-electrons, respectively. The reaction in aprotic acetonitrile initially yields the Mo(5+) complex **1-Mo(5+)**, but further Fc^+^ addition produces the previously reported Mo(4+) complex **2-H** bearing a protonated oxidized pterin. We propose the formation of **2-H** results from a one e^–^ oxidation of the pterin to yield a pterin radical that quickly undergoes intramolecular electron transfer to produce the reduced Mo(4+) ion and an oxidized pyranopterin of **2-H**. Additional Fc^+^ addition eventually leads to formation of **2-Mo(5+)**, causing a net 3e^–^ oxidation reaction where both the Mo and the pterin are oxidized. It is presumed that the aprotic nature of acetonitrile slows all proton transfer processes and increases the lifetime of the intermediate **2-H** such that it can be observed. Consistent with this speculation is the observation that oxidation of Mo(4+) is fast (<1 min) whereas pterin redox is slow (>7 h). We note the two biochemical redox partners, FeS clusters and FAD, share a relationship with the redox reagents used in this study, such that FeS clusters as 1 e- oxidants might function similarly to ferrocenium, whereas the proton dependent FAD+ redox partners may react similarly to DCIP.

It should be highlighted how the six-coordinate structure of **1** leads to unusual outcomes in this study. The use of the tridentate Tp* ligand has a significant role in stabilizing the complex as a whole and may allow access to species that otherwise would not be detected. Many oxo-Mo(4+) model complexes for Moco are five coordinate. This leaves an open coordination site that is occupied by a second oxo ligand when the Mo(4+) ion is oxidized by 2 electrons. Two strong π-donating oxo ligands are well-known to provide a stabilizing environment for Mo in its highest oxidation state.^79^ Likewise, in pyranopterin Mo enzymes, Mo oxidation during the catalytic cycle is always accompanied by Mo-oxo group formation. Here we observe that a two-electron oxidation of **1** does not lead to Mo(6+) but instead proceeds to a Mo(5+) oxidized pterin species. This difference must derive from the inability of **1** to form a dioxo complex due the inaccessibility of a hepta-coordinate [Tp*Mo^VI^O_2_(S_2_H_2_BMOPP]^−^ structure. This behavior is similar to what has been observed previously for thiol-inhibited MsrP, where the Mo(VI) state cannot be accessed due to its inability to form a *cis*-dioxo structure.^78^

## Conclusions

Our results demonstrate how the unique attributes of the pyanopterin dithiolene ligand structure provide Moco the capability of variable redox reactivity and further illustrate how the pyranopterin may be involved in both one- and two-electron processes. In addition, the results reveal how a protic environment can influence the outcomes of pterin redox reactions. Indeed, the protein environment surrounding Moco is diverse across these enzymes, exhibiting various patterns of H-bonding and direct interactions with redox partners such as FeS clusters and FAD.^16,76^ The nature of H-bonding may serve to select for a particular reaction pathway. The sum of results reported here, together with previous examples of dithiolene redox,^33,34,38^ reveal an enormous versatility which may explain the flexibility of the Mo-pyranopterin dithiolene motif to catalyze a wide variety of substrate transformations.

## Supporting Information Available

General and synthetic methods; NMR data and selected spectra (^1^H, COSY, and NOESY) for 1; FT-IR spectrum of **1**; DFT structure optimizations for **1**; ESI and HRESI mass spectra for **1**; EPR parameters for **1-Mo(5+)**, **2-Mo(5+)**, **3-Mo(5+)**, and Tp*MoO(bdt); absorption spectra for oxidation reactions and titrations of **1** (PDF)

## Abbreviations

PDT: pyranopterin dithiolene
Tp*: *tris*(3,5-dimethylpyrazolyl)hydroborate
BMOPP: 6-(3-butynyl-2-methyl-2-ol)-2-pivaloyl pterin
TEA: tetraethylammonium
bdt: 1,2-benzene dithiolate
