# Supplementary material for: Molybdenum Cofactor Model Reveals Remarkable Redox Activity at Both Molybdenum and the Pyranopterin Dithiolene Ligand
Source: J Am Chem Soc. 2025 Apr 28;147(18):15088–99. doi: 10.1021/jacs.4c17577 (PMC12063175; doi:10.1021/jacs.4c17577)

Molybdenum Cofactor Model Reveals Remarkable Redox Activity at Both Molybdenum and the Pyranopterin Dithiolene Ligand

Jinming Liu[a], Angelina Rogatch[a], Benjamin R. Williams[a], Chelsea Freer[a], Chiara Zuccoli[a], Jing Yang*[b], Martin L. Kirk*[b], Sharon J. Nieter Burgmayer*[a]

[a]Department of Chemistry, Bryn Mawr College, Bryn Mawr, Pennsylvania 19010, United States

[b]Department of Chemistry and Chemical Biology, The University of New Mexico, MSC03 2060, 1 University of New Mexico, Albuquerque, New Mexico 87131-0001, United States

Supporting Information

Table of Contents

Scheme S1. Reaction scheme to **1**, **2_O**, **2_P** and **1-Mo(5+)** page 2
Experimental Section page 3
Syntheses page 4
Redox Reactions page 5
Characterization
Figure S1. HRESI-MS of **1** page 6
Figure S2. FTIR of **1** page 7
Figure S3. DFT structure optimizations of **1** page 7
Table S1. EPR parameters for **1-Mo(5+)**, **2-Mo(5+)**, **3-Mo(5+)**, and Tp*MoO(bdt) Page 8
Figures S4-S10. ^{1}D, COSY and NOESY NMR of **1** pages 8-11
Figures S11. Structure of **1** compared to Precursor Z page 12
Redox Reactivity Studies
Air oxidation of **2**
Figure. S12 Air oxidation of **2** to **2-Mo(5+)** in methanol page 13
DCIP Oxidations of **1**
Figure S13 – EPR of DCIP oxidation of **1** page 13
Ferrocenium Oxidations of **1**
Figure S14. Titration of **1** with 0.2 to 3.5 eq Fc^+ page 14
Figure S15. Electronic absorption and EPR spectra after Fc+ addition to **1** page 14
Figure S16. Timestudy of 1 eq Fc^+ reaction with **1** page 15
Figure S17. ESI-MS of 1 eq Fc^+ reaction of **1** page 16
Figure S18. Absorption spectrum of **2-H** in ACN page 17
Figure S19. Timestudy of 1.2 eq Fc^+ added to **1** monitored by absorption spectroscopy over 24 hrs page 17
Figure S20. Exponential Rise Fit of Absorption Data for the Air Oxidation of **1** page 18

Scheme S1. Reaction scheme to **1**, **2_o**, **2_p** and **1-Mo(5+)**

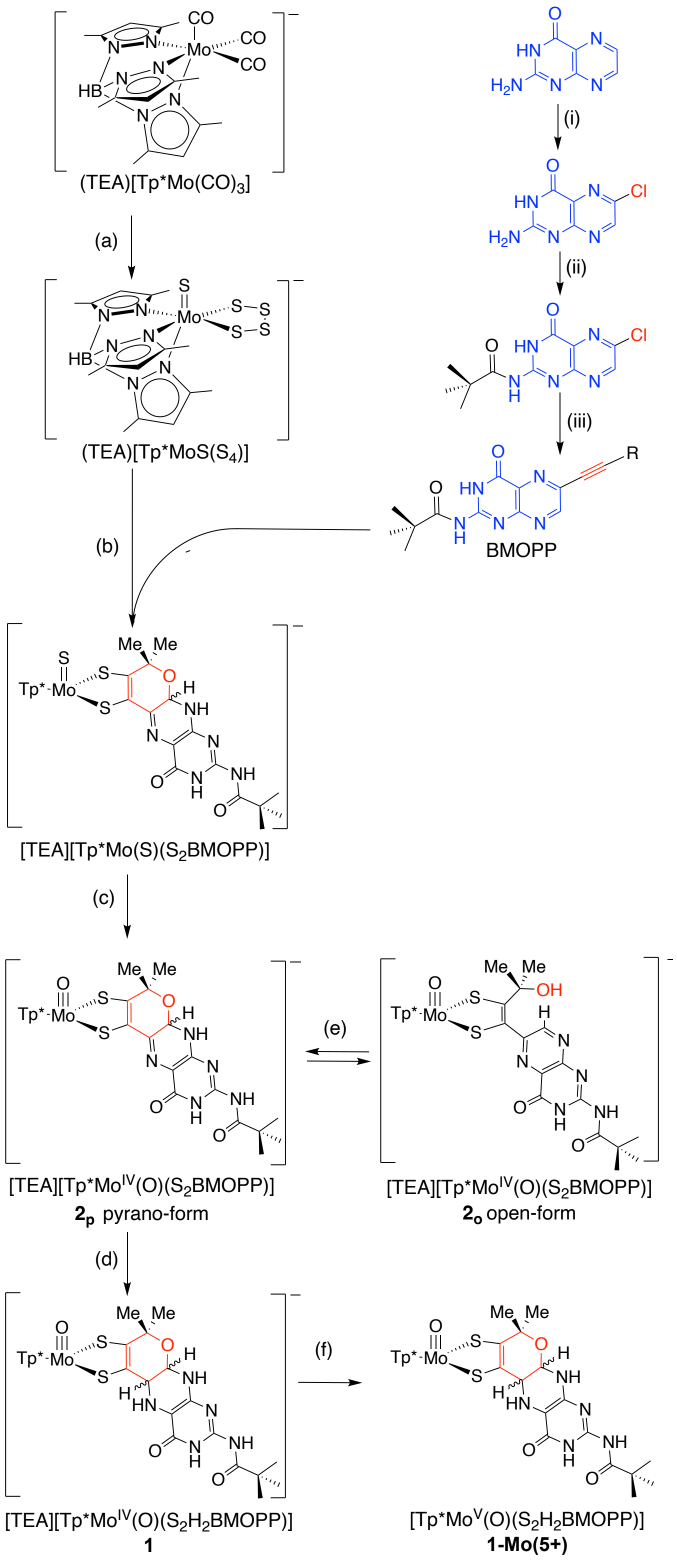

Experimental Section

Materials. All procedures and manipulations were carried out under a nitrogen atmosphere in a glovebox. Chemicals were purchased from Sigma-Aldrich, VWR International, Acros, and Alpha Aesar. Anhydrous solvents were purchased from Sigma-Aldrich stored under nitrogen. Synthesis of precursor [TEA][Tp*Mo^{IV}(O)(S_2BMOPP)] (**2**) was performed using the previously published procedure.[68] All glassware for synthesis and spectroscopy was dried in oven at 125 °C at least 24 hours, then cooled under high vacuum to prevent advantageous moisture from re-adhering to the glass.

NMR Spectroscopy. All NMR experiments were performed on a Bruker 400 MHz FT-NMR (^{1}H: 600 MHz). Chemical shifts are reported in parts per million (δ, ppm) and referenced to residual protic solvent resonances: (δ $CHCl_3$ = 7.26, CD_2HCN = 1.94, CD_2HOD = 3.31 ppm).

Fourier Transform Infrared Spectroscopy. Infrared spectra were obtained using a PerkinElmer Frontier FT-IR equipped with a Pike Industries GladiATR ATR stage on powdered solid samples.

Electronic Absorption Spectroscopy. Room temperature solution electronic absorption spectra were acquired using an Agilent 8453 spectrophotometer UV-vis-NIR spectrophotometer. The samples were prepared in the drybox dissolved in anhydrous acetonitrile and collected under a N_2 atmosphere in sealed quartz cells with a pathlength of 1 cm.

Mass Spectrometry. ESI-MS analyses were acquired at Bryn Mawr College using a Waters Micromass-ZQ mass spectrometer. High resolution mass spectrometric data was obtained from the University of Delaware Department of Chemistry and Biochemistry Mass Spectrometry Facility with the Orbitrap Q-Exactive mass spectrometer using an ion source of negative and positive mode electrospray ionization, which was coupled to an UltiMate 3000 UHPLC. Samples were direct-injected by syringe pump in LC-MS grade acetonitrile.

EPR Spectroscopy. Room temperature and 77K CW X-band (9.4 GHz) EPR spectra were collected using a Bruker ESP 300 spectrometer with associated Bruker magnet control electronics and microwave bridge. Low temperature spectra were collected using an Oxford Instruments ESR 910 liquid helium flow cryostat and an Oxford Instruments temperature controller (ITC 503). Compound **1-Mo(5+)** was generated in situ by dissolving **1** in anhydrous acetonitrile and adding 0.9 or 1 eq aliquots of ferrocenium hexafluorophsphate ($FcPF_6$) for the room temperature experiments, and in *n*-butyronitrile to form a glassy matrix for the low temperature experiments. All samples were prepared under a dry N_2 atmosphere to avoid any oxygen contamination and the addition of adventitious moisture. EPR spectral simulations were performed using EasySpin (version: 5.2.30) embedded in the Matlab (version: R2022a) platform.

Cyclic Voltammetry. Electrochemical analyses were performed under a nitrogen atmosphere using a BASi Epsilon-EC potentiostat with 0.1 M tetrabutylammonium perchlorate (TBAP) as the electrolyte in anhydrous acetonitrile, platinum working and auxiliary electrodes, and a Ag/AgCl reference electrode. All potentials reported are referenced to an internal ferrocene potential where E ($Fc^{+/0}$) is +440 mV vs. the Ag/AgCl electrode in ACN.

DFT Computations. Geometry optimizations and TDDFT computations on all diastereomers of **1** were performed at the density functional theory (DFT) level using the hybrid exchange-correlation functional (B3LYP) that were performed using the ORCA suite (v 4.2.1).[75] Valence triple-zeta polarization basis sets were employed, with def2-TZVP for the light atoms and def2-TZVPP for Mo and S atoms. EPR spin-Hamiltonian calculations on optimized structures of *RR*- and *SS*- **1-Mo(5+)** employed the zeroth order

relativistic approximation (ZORA) Hamiltonian for the relativistic correction and the old-ZORA-TZVP basis set for Mo, ZORA-def2-TZVPP basis set for S atom, and ZORA-def2-TZVP for all other elements. The solvation effect, conductor-like polarizable continuum model (CPCM), has been included for all geometry optimizations and spectroscopic property calculations.

Syntheses and Experimental Details

Synthesis of (TEA) [Tp*Mo^{IV}(O)(S_2H_2BMOPP)] (**1**). In the glovebox, (TEA)[Tp*Mo^{IV}(O)(S_2BMOPP)] (**2**) (153 mg, 0.160 mmol) was dissolved in 10 mL of anhydrous methanol forming an orange-yellow solution. KBH_4 (20 mg, 0.37 mmol, 2.25 eq) was dissolved in 4 mL of methanol. A 1.1 eq portion of the KBH_4 solution (2 mL) was transferred to the orange-yellow solution in the reaction flask. The reaction was stirred for 5 min at room temperature during which the solution color changed to lemon yellow. The reaction solution was evaporated in vacuo within the drybox to dryness. The brownish yellow residue was dissolved into minimal ACN (7 mL) and the solution was treated with 15 mL of 25% aqueous NH_4Cl and stirred (5 min), resulting in formation of white precipitate (KCl). The reaction solution was filtered with a syringe filter (1.0 micron) to remove the KCl precipitate which was washed with ACN (1 mL). The filtrate and washings were charged into a Schlenk flask, then evaporated to dryness in vacuo. The resulting residue was redissolved in 1.5 mL ACN, then added to 40 mL of diethyl ether with stirring to precipitate a yellow-gold solid. The yellow product (113 mg, 75%) was collected through vacuum filtration in a 15 mL fine fritted funnel and washed with diethyl ether. During the course of the borohydride reduction reaction of precursor **2**, formation of the product (TEA)[Tp*Mo^{IV}O(S_2H_2BMOPP)] **1** is observed by ESI-MS as m/Z = 806 in the negative ion mode, which corresponds to the addition of two hydrogen atoms to **2**. Unexpected difficulties encountered during the pterin reduction involve the partial cleavage of the pivaloyl group appended to the amino group at pterin C2. Incorporating the addition of aqueous ammonium chloride during reaction work-up prevented amide hydrolysis. The 1-electron oxidized complex **1-Mo(5+)** is generated in situ by addition of 1 eq ferrocenium hexafluorophosphate in acetonitrile.

^{1}H NMR (MeOH-d^4, 400MHz) 6.01 (s, 0.5H), 6.00 (s, 0.5H), 5.99 (s, 1H), 5.43 (s, 0.5H), 5.40 (s, 0.5H), 5.26 (d, 0.5H, J = 1.8 Hz), 5.20 (d, 0.5H, J = 1.8 Hz), 3.71 (d, 0.5H, J = 1.8 Hz), 3.56 (d, 0.5H, J = 1.8 Hz), 2.72 (s, 3H), 2.71 (s, 1.5H), 2.70 (s, 1.5H), 2.48 (s, 6H), 2.184 (s, 1.5H), 2.178 (s, 1.5H), 2.07 (s, 1.5H), 1.95 (s, 1.5H), 1.76 (s, 3H), 1.66 (s, 1.5H), 1.64 (s, 1.5H), 1.53 (tt, 24H, J = 1.3 Hz), 0.90 (s, 12H).

ESI-MS: M- m/z 806; ESI+MS: [M- + 2TEA+] m/z 1086.

HR-ESIMS: M- m/z 806.21070; calcd for $C_{31}H_{43}O_4N_{11}BMoS_2$: M- m/z 806.20935.

UV/vis (CH_3OH) (ε, M^{-1} cm^{-1}): 288 (14,900), 375 (10,120), 445 (7020) and 521 (2430).

FT-IR (cm^{-1}): 2975 (w), 2923 (w), 2524 (w), 1656 (m), 1619 (very weak), 1574 (very weak), 1542 (m), 1447 (m), 1413 (m), 1371 (m), 1208 (m), 1169 (m), 1093 (very weak), 1065 (w/m), 1035 (m), 1000 (very weak), ν(Mo≡O) 918 (s), 852 (m), 812 (w), 778 (s), 696 (m), 646 (m), 466 (m).

Redox Reactions

Redox titration with 2,6-dichlorophenolindophenol (DCIP).

(TEA)[Tp*Mo^{IV}(O)(S_2H_2BMOPP)] (2.8 mg, 3.0 µmol) and DCIP (13 mg, 48.5 µmol) were dissolved in 30 mL and 10 mL of methanol, respectively. An aliquot of the DCIP solution (0 - 1.75 eq) was added dropwise to 1 mL of the TEA[Tp*Mo^{IV}(O)(S_2H_2BMOPP)] solution, and the volume of each sample was diluted to 3 mL with MeOH. The cuvettes were capped in an inert atmosphere, and the UV-Vis absorbance was measured immediately upon sample preparation.

DCIP Time Study (UV-vis)

All samples were prepared in an inert atmosphere. (TEA)[Tp*Mo^{IV}(O)(S_2H_2BMOPP)] (2.8 mg, 3.0 µmol) and DCIP (13 mg, 48.5 µmol) were dissolved in 30 mL and 10 mL of methanol, respectively. A 3-mL aliquot of the oxidized stock solution was transferred to a screw top cuvette and removed from the glovebox for UV-vis absorbance measurements over 6 hours. To monitor the reaction, an aliquot of the DCIP solution (0-1.75 eq) was added dropwise to 1 mL of the TEA[Tp*Mo(O)(S_2H_2BMOPP)] solution. The reaction was monitored by absorption spectroscopy over 24-40 hours.

Redox titration with ferrocenium hexafluorophosphate ($Fc^+PF_6^-$).

Fc^+ Titration (UV-vis)

All samples were prepared in an inert atmosphere. TEA[Tp*MoIV(O)(S_2H_2BMOPP)] (4.6 mg, 4.9 µmol) and ferrocenium hexafluorophosphate (6.6 mg, 19.9 µmol) were dissolved in 16 mL and 10 mL of ACN, respectively. An aliquot of the Fc^+ solution (0-3 eq) was added dropwise to 1 mL of the 806 solution, and the sample volume was diluted to 3 mL with ACN. The cuvettes were capped in an inert atmosphere, and the UV-Vis absorbance was measured immediately upon sample preparation.

Fc^+ Time Study (UV-vis)

All samples were prepared in an inert atmosphere. TEA[Tp*Mo(O)(S_2H_2BMOPP)] (2.3 mg, 2.46 µmol) and ferrocenium hexafluorophosphate (17.1 mg, 51.7 µmol) were dissolved in 25 mL and 10 mL of either ACN or MeOH in respective volumetric flasks. A 3-mL aliquot of the oxidized stock solution was transferred to a screw top cuvette and taken out of the glovebox for UV-vis absorbance measurements over 6 hours. An aliquot of the Fc^+ solution was added dropwise to the TEA[Tp*Mo (O)(S_2H_2BMOPP)] solution. The reaction was monitored over 24-40 hours.

Figure S1. HRESI-MS of 1. (top) Experimental ESI-MS(-) spectrum for the molecular anion $C_{31}H_{43}O_4N_{11}BMoS_2$. (bottom) Detail showing isotopic pattern.

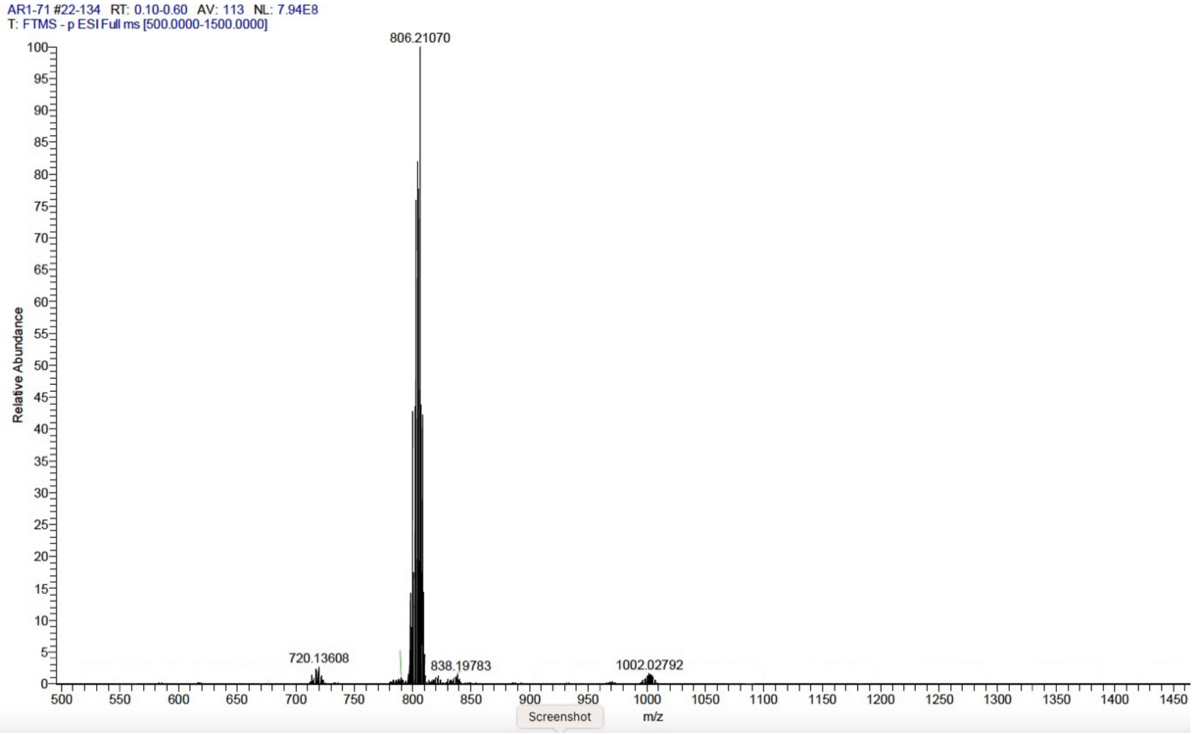

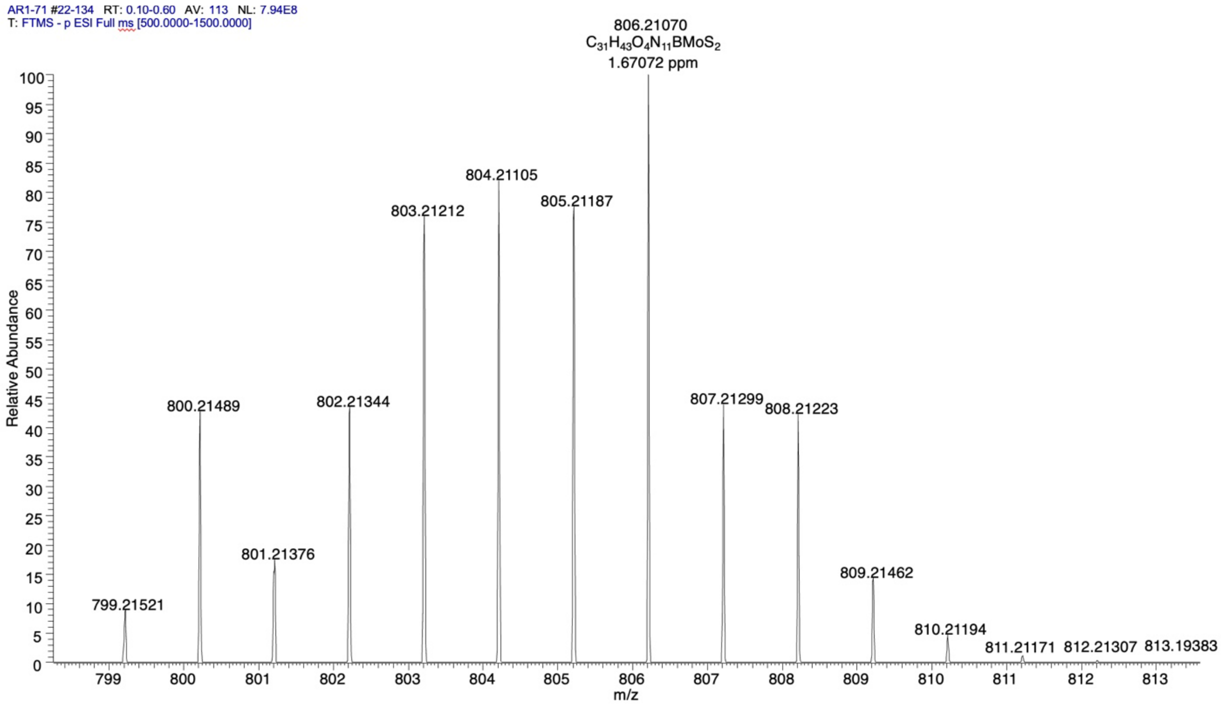

Figure S2. FTIR of 1.

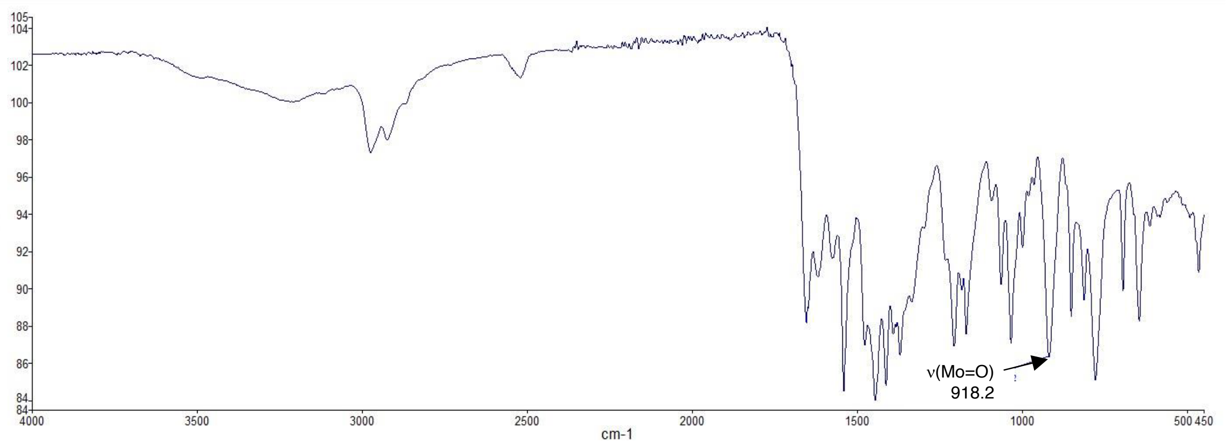

Figure S3. DFT optimized structures of *R,R-*, *S,S- R,S-* and *S,R-* diastereomers of 1. The structure of *R,R*-**1** possesses the lowest optimized energy.

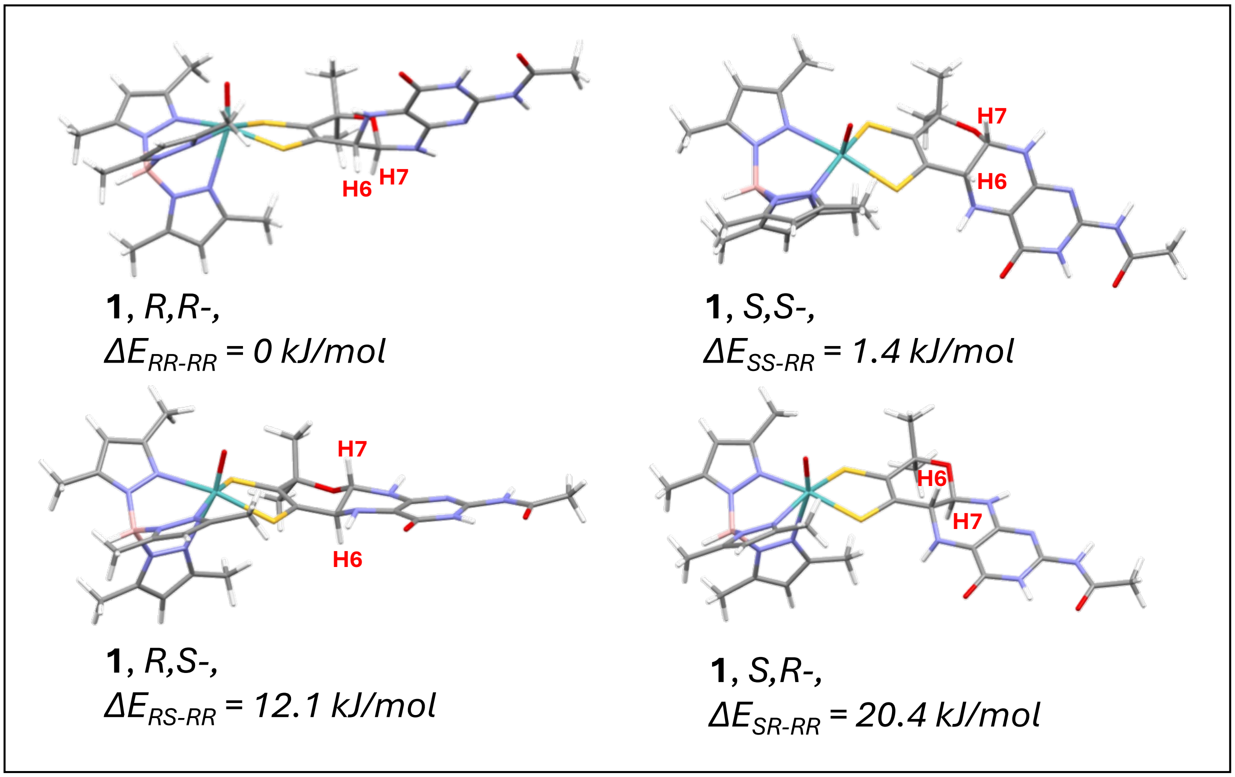

Table S1. Experimental EPR spin Hamiltonian parameters for **1-Mo(5+)**, **2-Mo(5+)**, **3-Mo(5+)**, and Tp*MoO(bdt) and computed spin Hamiltonian parameters for **1-Mo(5+)** *R,R*- and *S,S*-diastereomers.

	g_1	g_2	g_3	$g_{iso/ave}$	A_1	A_2	A_3	$A_{iso/ave}$	α	β	ϒ
					Unit: MHz				Unit: degree		
***RT*, 1-Mo(5+)**				1.9695				101.3			
***77K*, 1-Mo(5+)**	2.0035	1.9733	1.9345	1.9704	69	71	164	101.3	0	36	0
Calc, *R,R*-**1-Mo(5+)**	2.0098	1.9787	1.9396	1.9760	54.1	54.3	156	88.20	1.7	43.3	-2.5
Calc, S,S-**1-Mo(5+)**	2.0080	1.9779	1.9406	1.9755	54.6	55.4	157.6	89.18	1.5	45.0	-3.7
77K, **2-Mo(5+)**	2.0022	1.9716	1.9335	1.9692	62.1	69.9	174	102.0	-14.5	48.5	19.5
77K, **3-Mo(5+)**	2.0032	1.9775	1.9385	1.9730	78	62.1	158	99.3	-2.0	31	13
***77K*, Tp*MoO(bdt)**	2.004	1.972	1.934	1.970	75	78	180	101.0	0	45	0

Figure S4. **Labeling schemes and DFT optimized structures used for NMR assignments of 1.**

Labeling Scheme for ^{1}H NMR proton assignments for (left) *RR*- and (right)*SS*- diastereomers of **1**

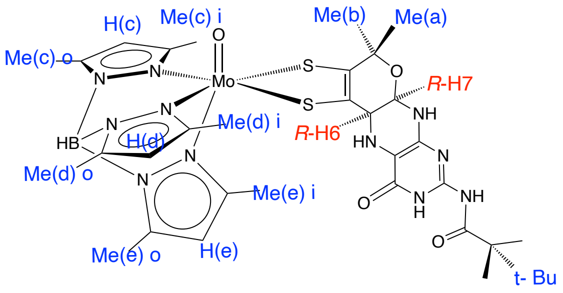

R,R-1

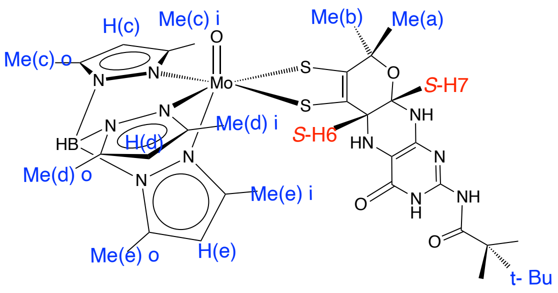

S,S-1

DFT optimized structures for (left) *R,R*- and (right) *S,S*- diastereomers of **1**

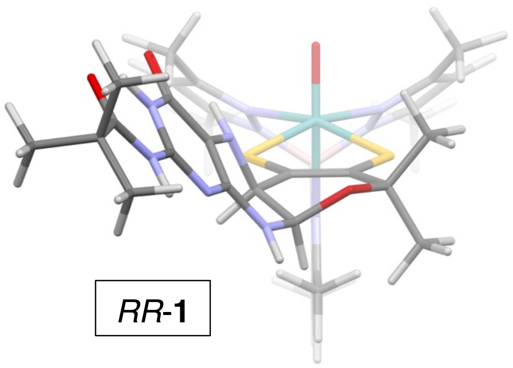

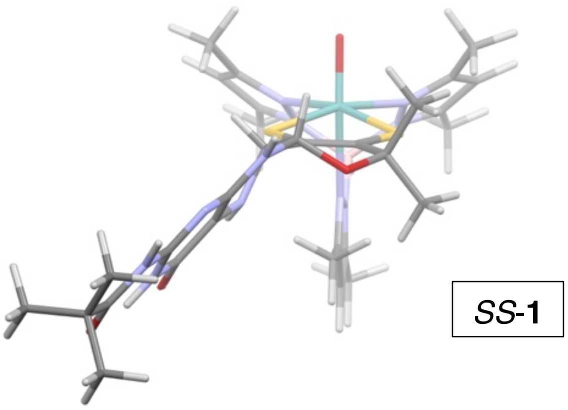

Figure S5. (a) (top) Full 1-D ^{1}H NMR spectrum of **1** in MeOH-d$_4$.
(bottom) Expanded spectrum with proton assignments

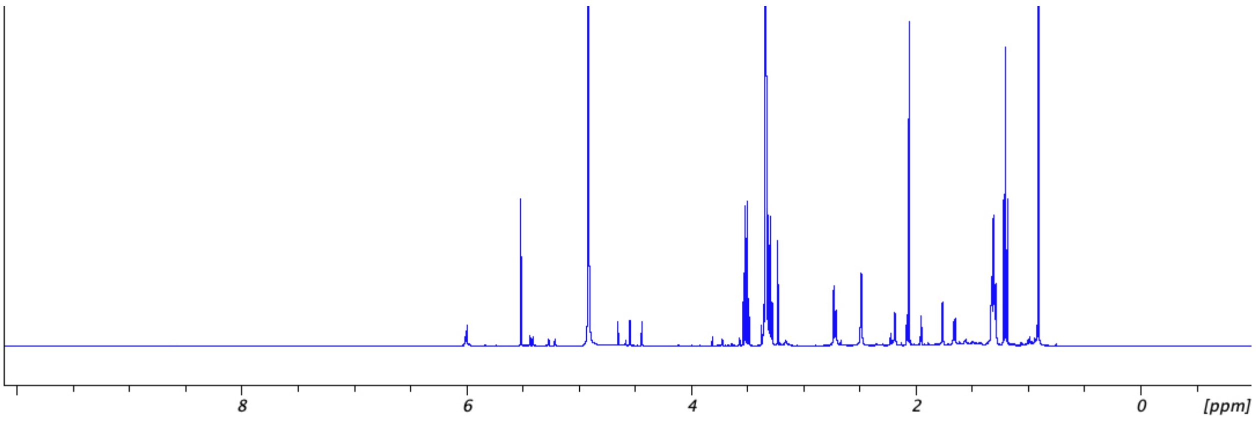

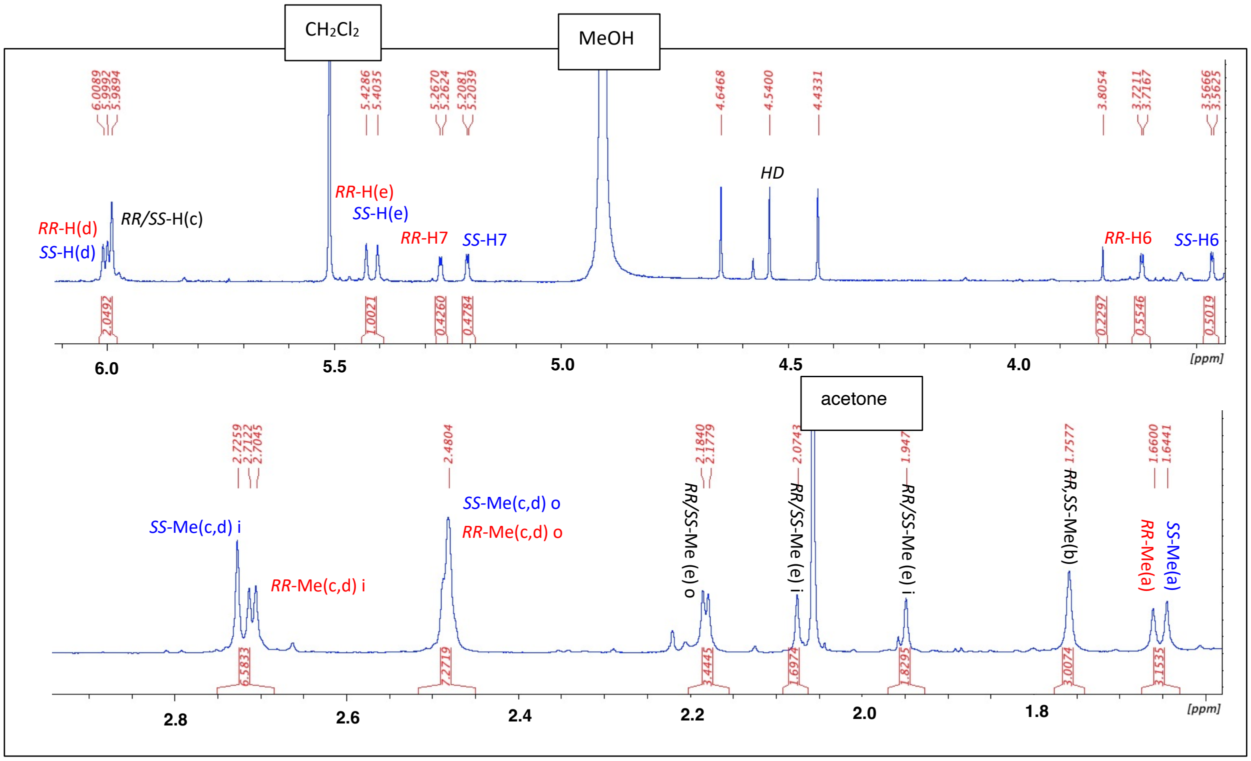

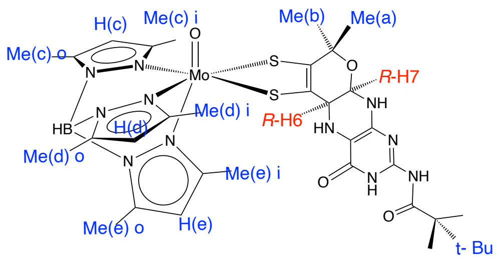

RR-**1**

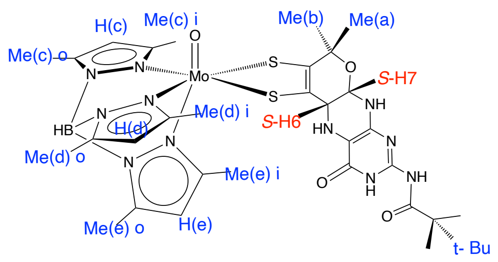

SS-**1**

Figures S6-S8. Selected COSY and NOESY of 1

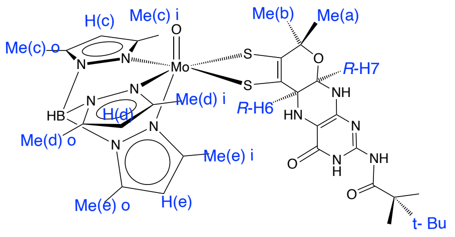

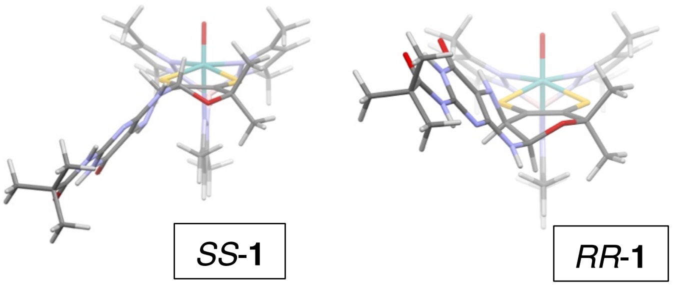

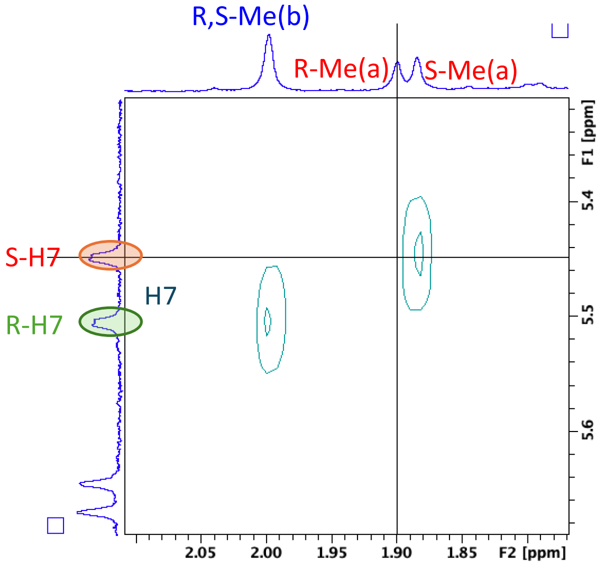

Figure S6. NOESY section highlighting H7 interactions with pyran Me(a) and Me(b) groups. (See labeled structure above). This was used to distinguish *R*- vs *S*- H7 identities. DFT calculations show that only *S*- H7 has through-space interactions with Me(a). Therefore the red highlighted resonance is assigned as *S*- H7 and green highlighted resonance is assigned as *R*-H7.

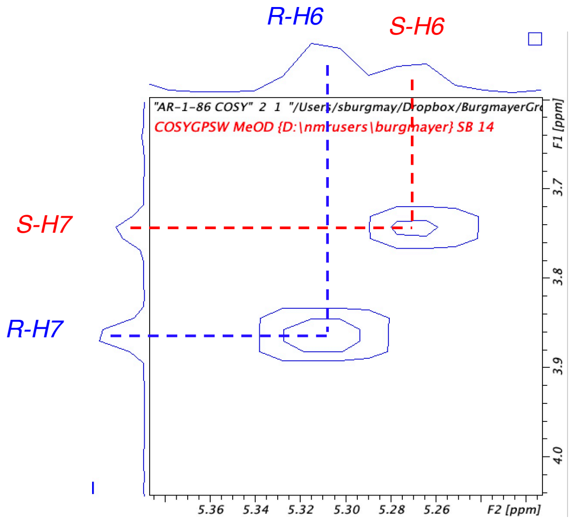

Figure S7. COSY section highlighting R- and S-H7 interactions with R- and S-H6, which allows assignment of H6 resonances.

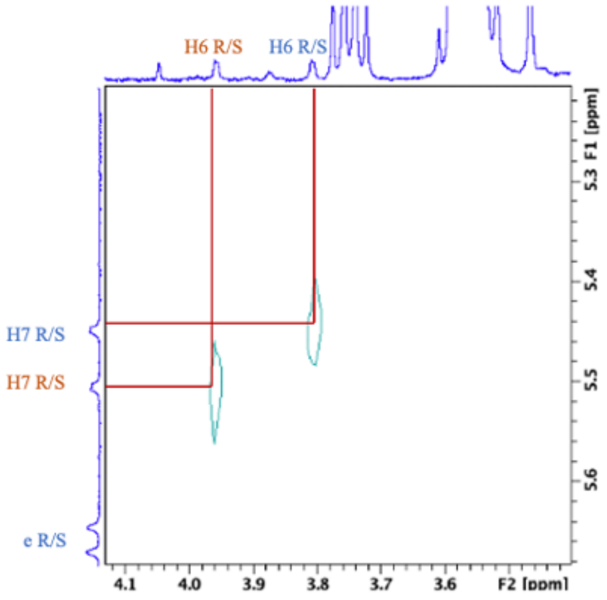

Figure S8. NOESY section highlighting R- and S-H7 interactions with R- and S-H6.

Figure S9-S10. Selected NOESY spectra of 1

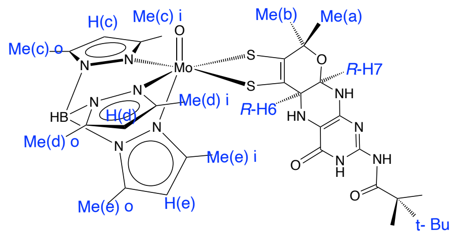

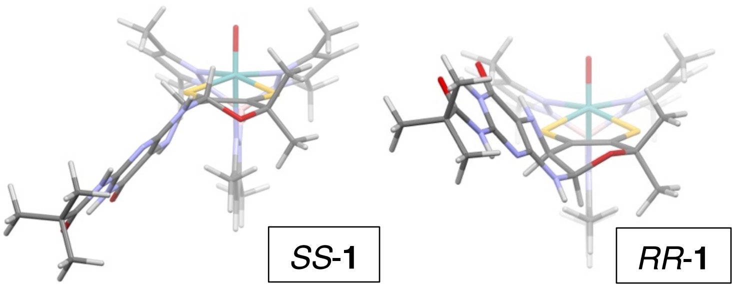

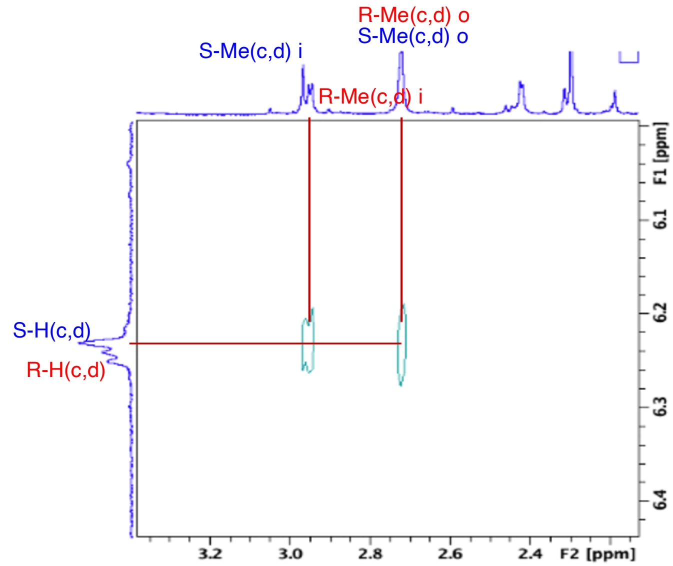

Figure S9. NOESY section highlighting interactions between protons and methyl groups on pyrazolyl rings c and d.

Only Me(c) have through-space interactions with H(c) hydrogen, and likewise Me(d) only have through-space interactions with H(d)
The 2.7 and 2.9 peaks are a combination of both Me(c) and Me(d).

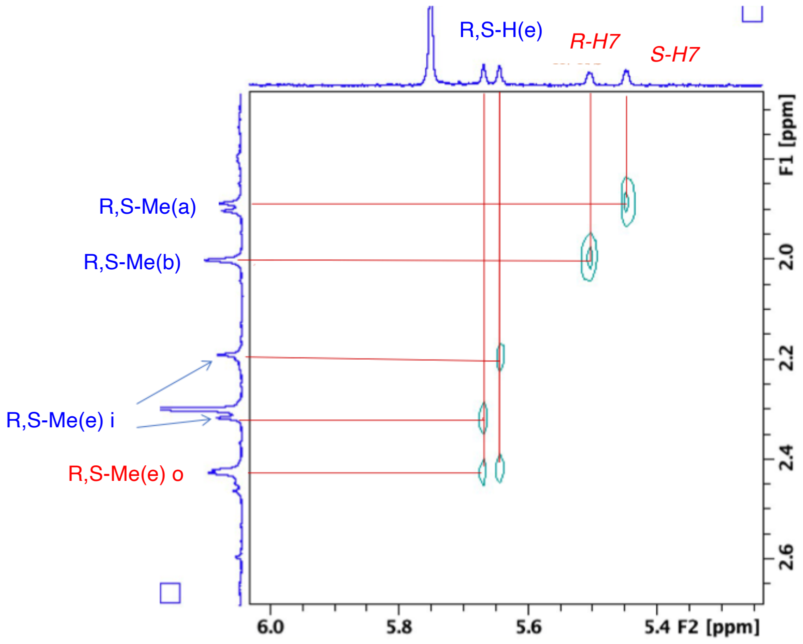

Figure S10. NOESY section highlighting interactions between *R*-H7, *S*-H7, pyran Me(a) and Me(b), and pyrazolyl Me(e) and H(e). (See labeled structures above).

Figure S11. **Comparison of structures of 1 and Precursor Z where green highlighted protons correspond to H6 and H7 in 1.**

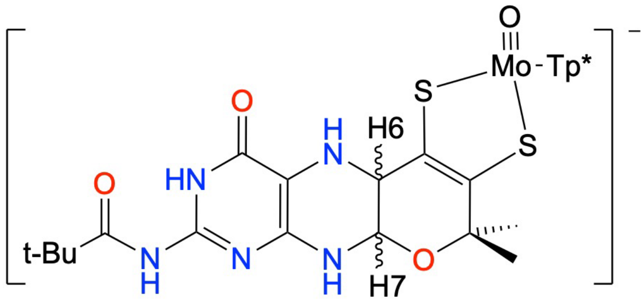

(TEA)[Tp*MoO(S_2H_2BMOPP)]
1

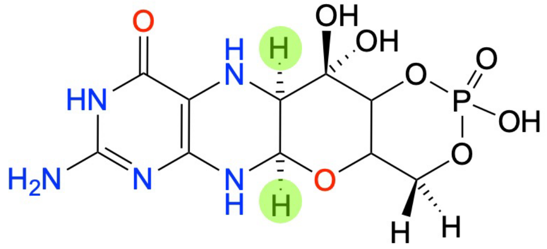

Precursor Z

Figure. S12. Air oxidation of 2 to 2-Mo(5+) in methanol.

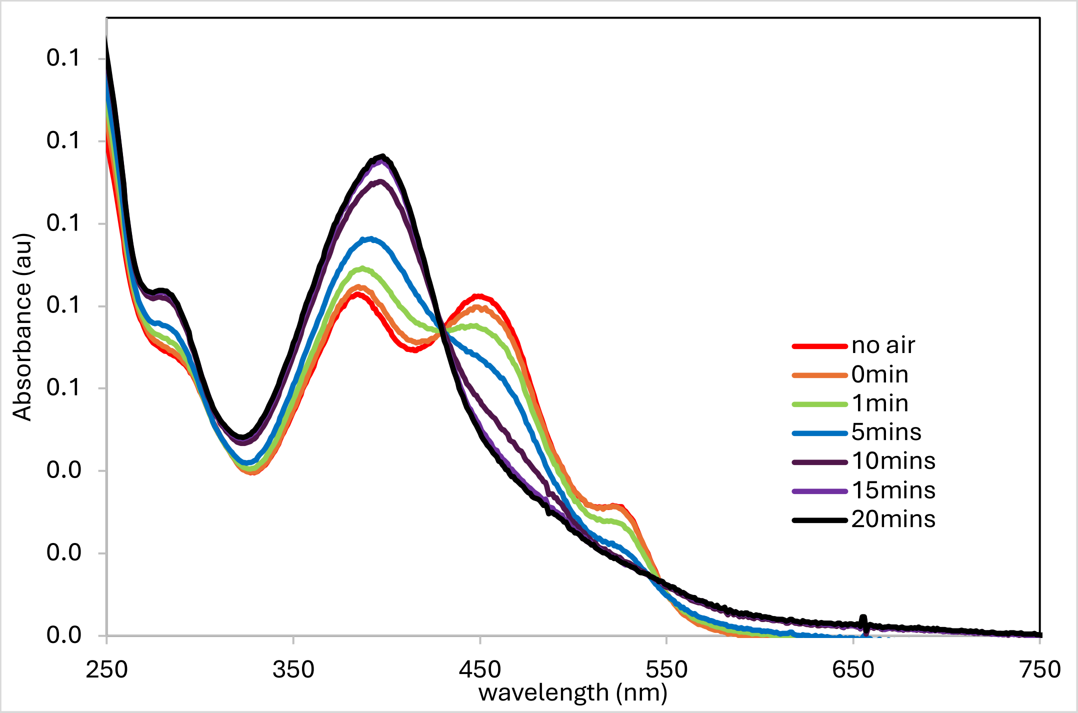

Figure S13. EPR of DCIP oxidation of 1.

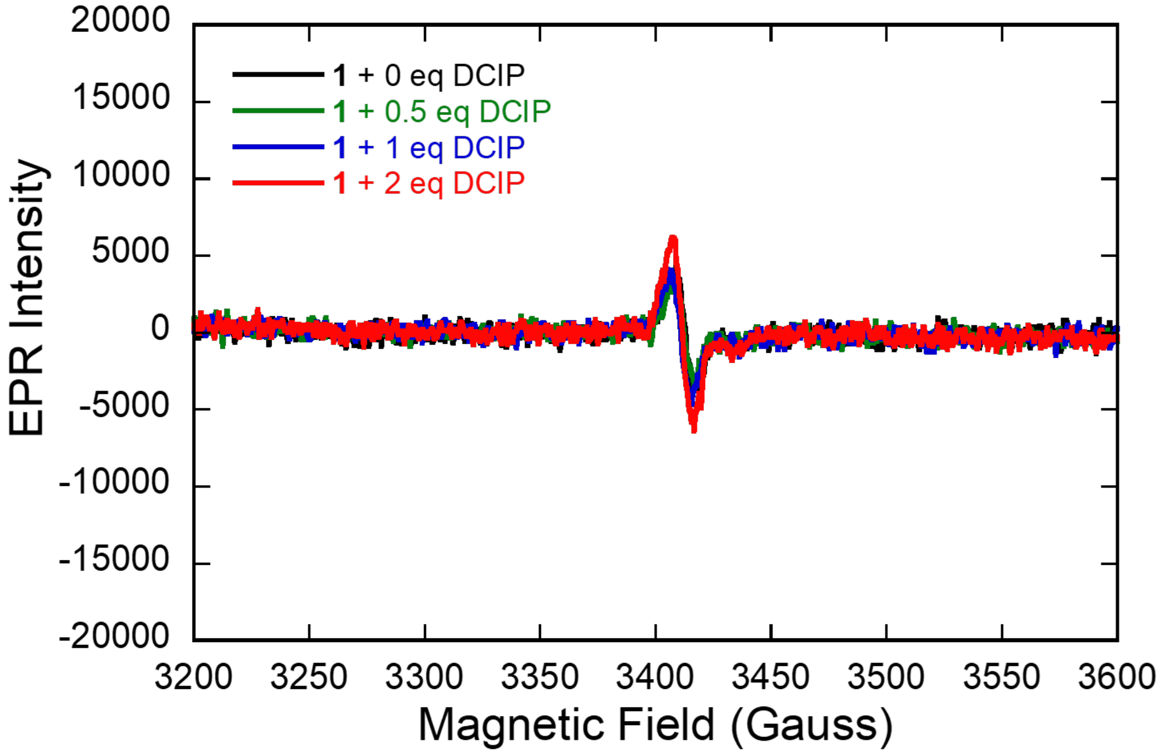

Figure S14. Titration of 1 with 0.2 to 3.5 eq Fc+ in acetonitrile.

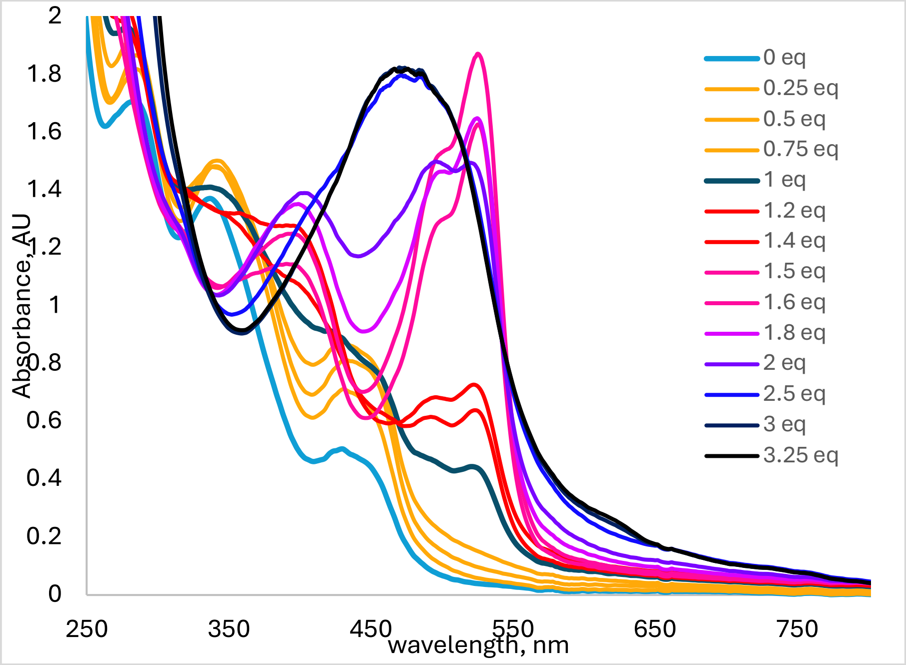

Figure S15. (Top) Electronic absorption spectra of 1 before (black line) and after (red line) addition of 1 eq Fc+ in acetonitrile forming 1-Mo(5+). (Bottom and inset) Room temperature EPR spectra of 1 before and after adding 1 eq Fc+ and the change over time.

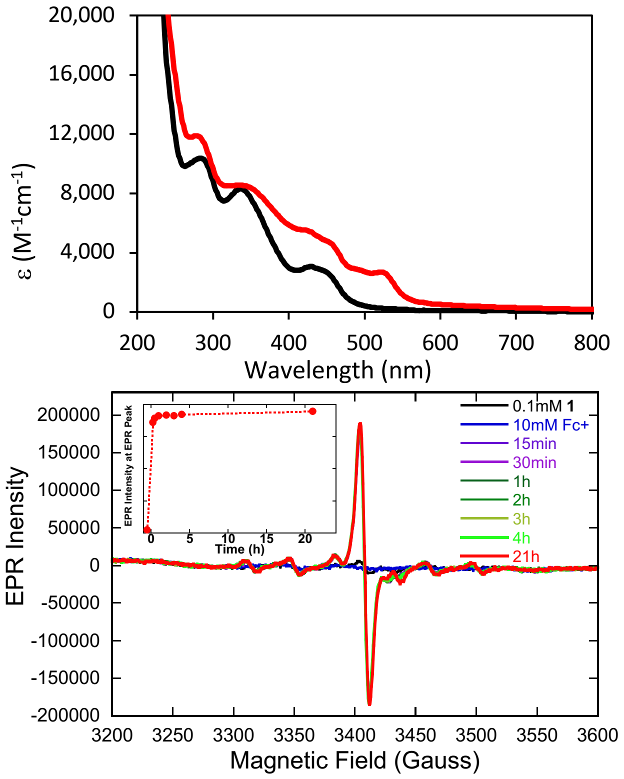

Figure S16. Timestudy of 1 eq Fc+ reaction with 1 in acetonitrile.

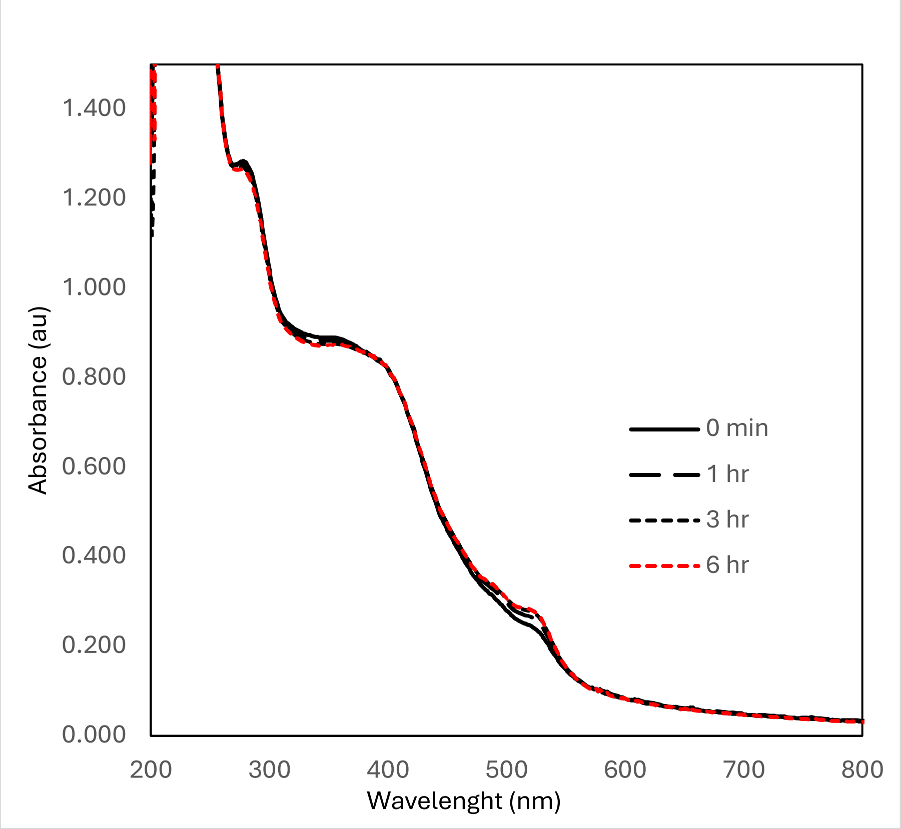

Figure S17. Titration of 1 by Fc^{+} (0-2.5 eq) in acetonitrile monitored by +ESI-MS.

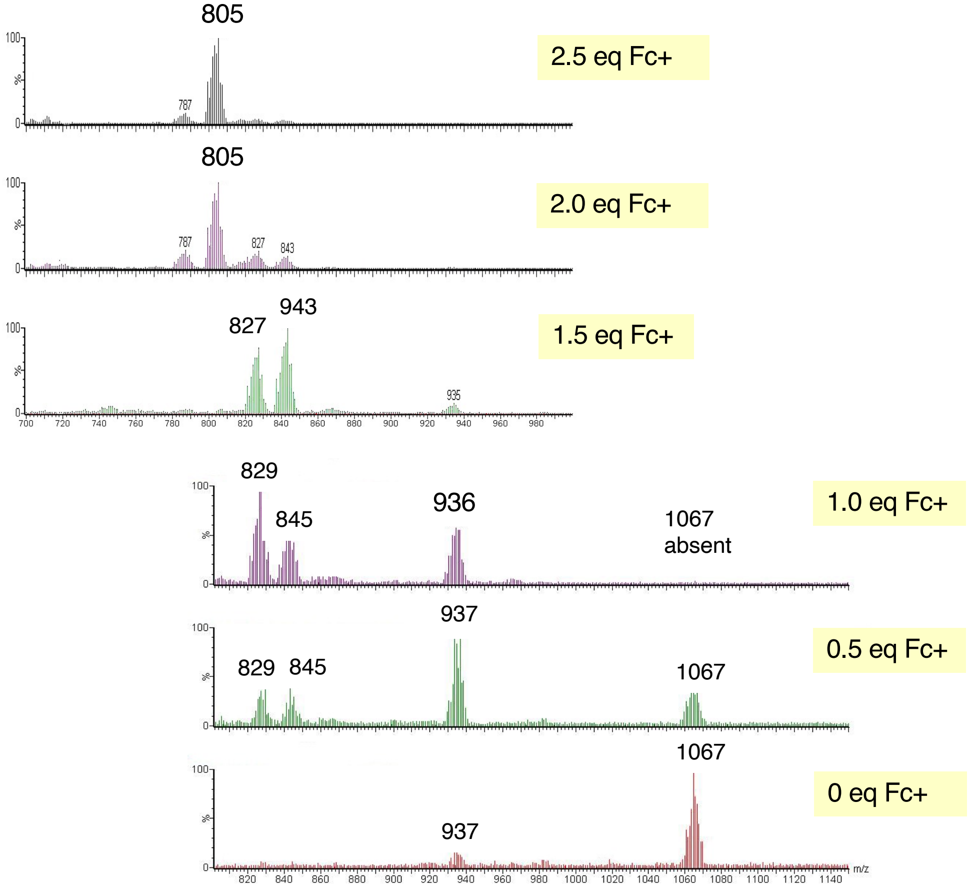

**m/Z.**	**species identity**
1067	= {M*(4+) + 2TEA^{+}}
845	= {M*(5+) + K^{+}}
943	= {M(5+) + K^{+}
937	= {M*(4+) + H^{+} + TEA^{+}}
936	= {M*(5+) + TEA^{+}}
829	= {M*(5+) + Na^{+}}
827	= {M(5+) + Na^{+} }

M* = m/Z 806, **1**
M = m/Z 804, **2**

Figure S18. Absorption spectrum of 2-H in acetonitrile.

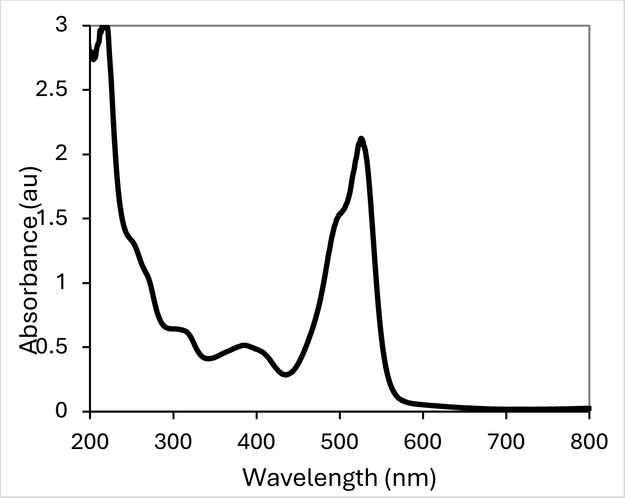

Figure S19. Time study of 1.2 eq Fc^{+} added to 1 monitored by absorption spectroscopy over 24 hrs.

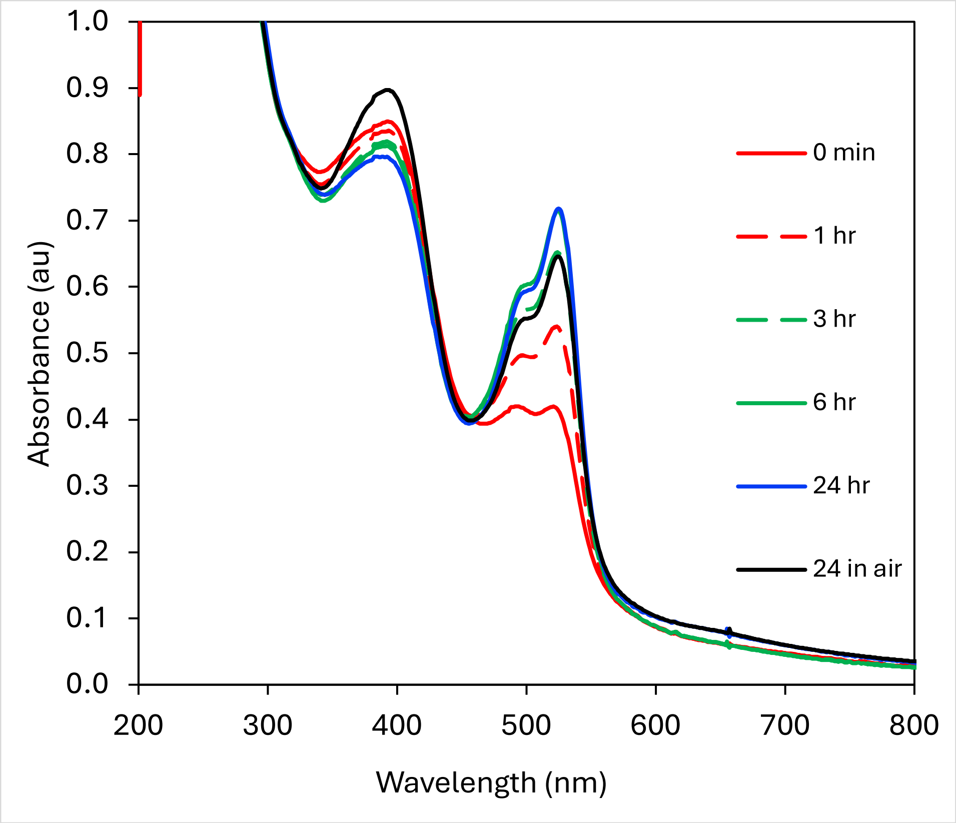

Figure S20. Exponential Rise Fit of Absorption Data for the Air Oxidation of 1

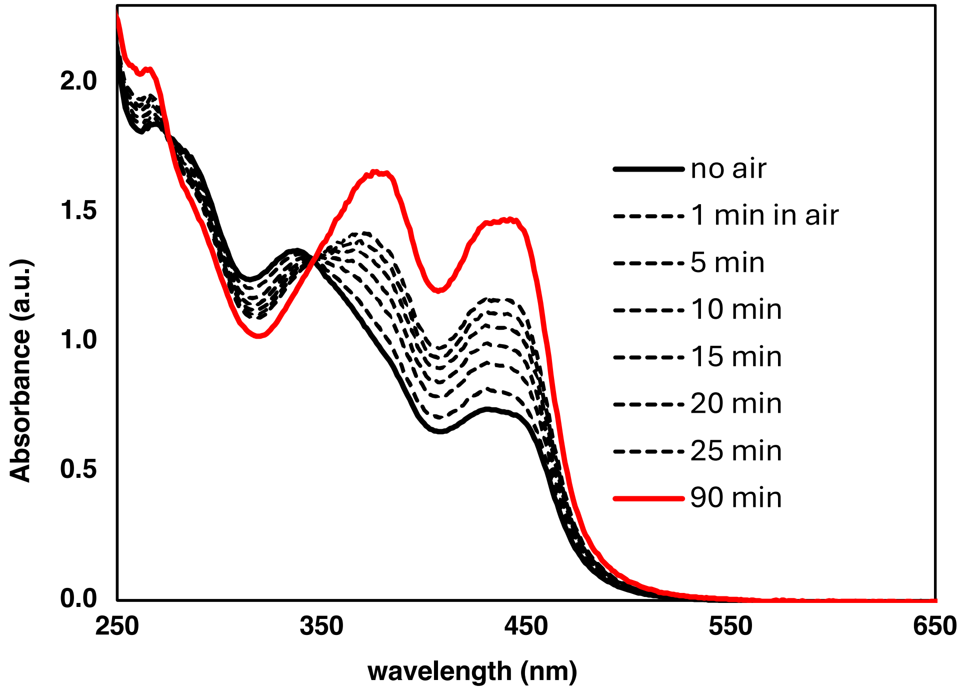

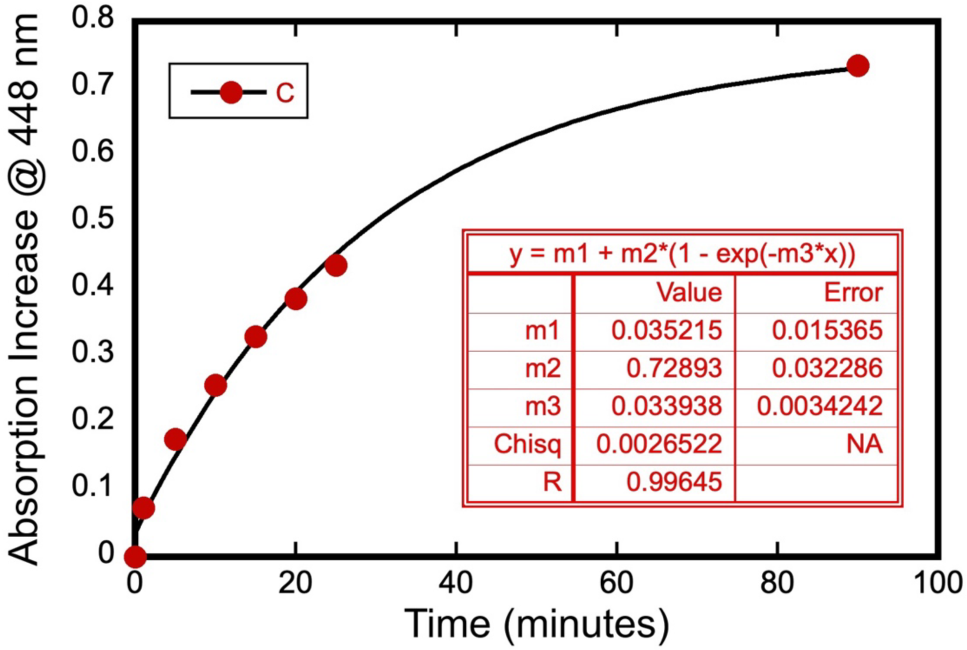

Supplement: Supplementary file 1 — ja4c17577_si_001.pdf [file ja4c17577_si_001.pdf]
